# It's about time: Feeding competition costs of sociality are affected more by temporal characteristics than spatial distribution

**DOI:** 10.1002/ece3.11209

**Published:** 2024-04-16

**Authors:** Marcy Ekanayake‐Weber, Namita Mathew, Deanna Cunha, Nathanael Payen, Volker Grimm, Andreas Koenig

**Affiliations:** ^1^ Department of Anthropology Stony Brook University Stony Brook New York USA; ^2^ Interdepartmental Doctoral Program in Anthropological Sciences Stony Brook University Stony Brook New York USA; ^3^ Department of Computer Science Stony Brook University Stony Brook New York USA; ^4^ Department of Ecological Modeling Helmholtz Centre for Environmental Research – UFZ Leipzig Germany; ^5^ Graduate Program in Ecology and Evolution Stony Brook University Stony Brook New York USA

**Keywords:** agent‐based model, foraging, group living, resource availability, resource distribution, resource quality

## Abstract

For most herbivorous animals, group‐living appears to incur a high cost by intensifying feeding competition. These costs raise the question of how gregariousness (i.e., the tendency to aggregate) could have evolved to such an extent in taxa such as anthropoid primates and ungulates. When attempting to test the potential benefits and costs, previous foraging models demonstrated that group‐living might be beneficial by lowering variance in intake, but that it reduces overall foraging success. However, these models did not fully account for the fact that gregariousness has multiple experiences and can vary in relation to ecological variables and foraging competition. Here, we present an agent‐based model for testing how ecological variables impact the costs and benefits of gregariousness. In our simulations, primate‐like agents forage on a variable resource landscape while maintaining spatial cohesion with conspecifics to varying degrees. The agents' energy intake rate, daily distance traveled, and variance in energy intake were recorded. Using Morris Elementary Effects sensitivity analysis, we tested the sensitivity of 10 model parameters, of which 2 controlled gregarious behavior and 8 controlled food resources, including multiple aspects of temporal and spatial heterogeneity. We found that, while gregariousness generally increased feeding competition, the costs of gregariousness were much lower when resources were less variable over time (i.e., when calorie extraction was slow and resource renewal was frequent). We also found that maintaining proximity to other agents resulted in lower variance in energy intake when resources were more variable over time. Thus, it appears that the costs and benefits of gregariousness are strongly influenced by the temporal characteristics of food resources, giving insight into the pressures that shaped the evolution of sociality and group living, including in our own lineage.

## Graphical abstract

We developed simulations in which primate‐like agents foraged on a variable resource landscape while maintaining spatial cohesion with conspecifics to varying degrees. It appears that the costs and benefits of foraging socially are strongly influenced by the temporal characteristics of food resources, giving insight into the pressures that shaped the evolution of sociality and group living, including in our own lineage.

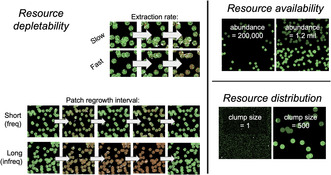

## 1 INTRODUCTION

Gregariousness, the behavioral tendency to tolerate and/or affiliate with conspecifics, can vary from near‐complete avoidance in certain species (e.g., pumas, Elbroch et al., 2017), to obligate sociality that creates large permanent groups in others (e.g., female elephants, de Silva & Wittemyer, 2012; eusocial insects, Nowak et al., 2010), with much variation and flexibility in between (e.g., otters, mice; Lodé et al., 2020; Schradin et al., 2020). Before considering why groups of a particular size form, which is likely to be influenced by many different factors, it is important to consider from an individual's perspective why an animal would coordinate its movement with conspecifics, i.e., what benefits or costs (or both) an individual faces when tolerating conspecifics nearby (Alexander, 1974; Miller, 1922). It has long been recognized that the proximity to conspecifics often appears to provide fitness benefits, or conversely, that low population density can lead to population extinction (Allee, 1931, 1938; Stephens et al., 1999). Reducing vulnerability to predation (Krause & Ruxton, 2002) is thought to be the main adaptive benefit of gregariousness (Alexander, 1974; Jarman, 1974; van Schaik, 1983). Gregariousness can also facilitate cooperative hunting (Bailey et al., 2013) or cooperative breeding (Canestrari et al., 2008; Russell et al., 2003), though these benefits are restricted to a small number of carnivore, rodent, and avian taxa, wherein associates are also often closely genetically related (Briga et al., 2012; Cornwallis et al., 2009).

Still, many gregarious taxa neither cooperatively hunt nor cooperatively breed, and also aggregate with non‐kin, such as most monkeys and apes (hereafter, “anthropoid primates”) and many artiodactyls and equids (hereafter, “ungulates;” Lukas & Clutton‐Brock, 2011, 2020). These herbivores are expected to experience feeding competition from proximity to conspecifics, and little foraging benefits from gregariousness (Janson, 1988; van Schaik, 1983) because they exploit mainly stationary, depletable, usurpable (Isbell & Young, 2002), and slow‐renewing food resources. In contrast, some researchers have hypothesized that foraging in association with conspecifics may be more efficient than foraging solitarily in overlapping home ranges (Altmann, 1974; Clark & Mangel, 1986; Cody, 1971; Miller, 1922). Rather than searching through areas that another conspecific has already depleted, foragers can coordinate movement to save time (Miller, 1922). As an additional benefit, the search area of multiple individuals is larger than that of a single forager, which should increase the frequency of encountering new food patches (Clark & Mangel, 1984; Georgiou et al., 2022). Thus, through information sharing, gregarious individuals may have higher foraging success, or their energy intake may be less variable (Clark & Mangel, 1986; Krause & Ruxton, 2002). However, data supporting these ideas are mixed (Rieucau & Giraldeau, 2011). Especially for anthropoid primates, few data support the idea that gregariousness provides foraging benefits (Janson, 1992), warranting further study.

To test the potential costs and benefits of different foraging patterns, some researchers have employed an agent‐based modeling approach. These studies found that group foraging tended to decrease energy intake and increase distance traveled, but also led to lower *variance* in energy intake (Beauchamp, 2005; Ruxton, 1995). In fact, avoiding a shortfall in energy intake may be more important than maximizing average foraging efficiency (Clark & Mangel, 1984; Marshall & Wrangham, 2007), so lower variance in food intake may be a key fitness advantage to gregariousness. However, there have been two key limitations of previous social foraging models (e.g., Beauchamp, 2005; Port et al., 2020; Ruxton, 1995). First, most models treated sociality as a dichotomous variable, contrasting only “solitary” foragers and “group” foragers, without recognition of the range of variation of gregariousness possible (e.g., Ruxton, 1995). Second, most models' resource landscapes were extremely simple, but the costs and benefits of gregariousness are likely highly contingent on a combination of resource and forager characteristics that impact resource competition (Koenig & Borries, 2006; Macdonald & Johnson, 2015). This variation cannot be accurately captured by broad dietary categories such as “frugivore”/“folivore,” or “clumped”/“uniform,” for these categories fail to explain much resource variation (Coiner‐Collier et al., 2016; Janson, 1988). Thus, past models (both agent‐based and verbal) could not represent specific resource characteristics that might impact the efficiency of gregarious foraging. Further investigation must consider various resource characteristics to meaningfully test how gregariousness influences feeding competition.

Furthermore, it is unclear which resource characteristics may be most important for influencing the costs and benefits of gregariousness because there are two contrasting views of which food resource characteristics most influence feeding competition (spatial vs temporal). Many have emphasized the role of resource distribution, i.e., “clumpedness” (Koenig & Borries, 2006), which describes how resource patches are located relative to each other on the landscape, on a spectrum from highly clumped/patchy to uniform or random. Resource distribution has been considered one of the most important in influencing resource competition (Chapman et al., 1995) since the grouping of resources might drive contest competition (Sterck et al., 1997), increase local animal density, or incentivize cooperative resource defense (Wrangham, 1980). The size of clumps and the distance between them is thought to determine travel costs for foragers (Chapman et al., 1995). Resource distribution also varies at multiple scales, i.e., patch size (smaller scale, typically a single feeding site) as compared to clump size (larger scale, with a clump being multiple patches grouped in space), and it is dependent on the spacing and the size of foragers relative to plant characteristics (Koenig & Borries, 2006). Given this complexity, the definitions of “clumped” or “patchy” have often been vague and contradictory (Isbell & Young, 2002). As part of this confusion, fruits have been assumed to be clumped, while leaves are assumed to be uniformly distributed (as implied in van Schaik, 1989), an assertion that has been frequently called into question (e.g., Koenig, 2000; Koenig et al., 1998; Snaith & Chapman, 2007).

Alternatively, others have characterized the variation in feeding competition as mainly resulting from temporal characteristics impacting the depletability of food (i.e., food site depletion time, FSDT; Isbell & Young, 2002). Depletability is affected by how quickly energy can be extracted from the resource (hereafter, extraction rate), as well as how quickly resources renew. Depletability may also be related to the frugivore‐folivore dichotomy often cited in the primate literature, since mature fruits and leaves may have different mechanical and chemical properties (Berthaume, 2016; but see Snaith & Chapman, 2005, 2007; Coiner‐Collier et al., 2016). Fruits are generally faster to consume, digest, and deplete, while gaining the same amount of energy from leaves may require more chewing, digestion time, and fermentation (Chapman et al., 2012). However, there are resources other than leaves that require handling and processing, including fruits with protective anatomical features, seeds or nuts that require extraction, underground storage organs requiring digging, or termite mounds that require tool use (Chapman et al., 2012; Fragaszy et al., 2004, 2023; Jarvey et al., 2018; Koops et al., 2013; Liu et al., 2009; Truppa et al., 2019). The Jarman‐Bell principle also posits that the fiber content of food will have a large effect on feeding competition because fiber increases chewing, fermenting, and digestion time, slowing down energy extraction and resource depletion (Bell, 1971; Geist, 1974; Jarman, 1974). Thus, higher fiber content of food may allow larger aggregations without much feeding competition cost (Bell, 1971; Geist, 1974; Jarman, 1974). Renewal has also been identified as an important factor in determining whether foraging with conspecifics has low enough costs to allow permanent groups to form (Macdonald & Johnson, 2015; Waser, 1981).

Finally, it bears emphasizing the distinction between gregariousness, which this study is directly investigating, and group size, which this study is only indirectly addressing. Gregariousness is the individual behavioral tendency to tolerate and/or be attracted to conspecifics, whereas group size is the emergent outcome of multiple behavioral and landscape factors that keep a set of individuals in proximity over a longer period. Compared to solitary living, being gregarious to any extent means compromising movement autonomy (Conradt & Roper, 2010), as well as likely sharing resources, whereas group size may only alter the degree of these costs. Therefore, the shift from avoiding conspecifics to being gregarious is fundamentally different from changes in group size, though the two have been conflated (e.g., van Schaik, 1983). This study focused on the foraging costs of gregariousness itself (i.e., the effect of individual behavior on individual foraging outcomes under different conditions) before attempting to investigate the more complex relationship between resource characteristics, group size, and foraging outcomes. A follow‐up study, where group size was more directly addressed, is currently in preparation.

In order to more fully investigate the impact of the interactions between resource characteristics and gregariousness on foraging success, we developed an agent‐based model of herbivorous animal foraging which includes aspects of gregarious behavior as well as complex (plant food) resource characteristics. Each parameter could be manipulated independently, allowing us to isolate which factors and interactions were most influential. We sought to answer two questions: (1) how do gregarious behaviors interact with particular resource characteristics to affect foraging? and (2) in what circumstances might gregariousness have very low costs, or even a feeding benefit? Based on the existing literature, we expected the following relationships:

*Prediction 1*: Across most resource distributions, greater gregariousness will be associated with higher foraging costs (i.e., lower energy intake, longer daily distances traveled, or both).
*Prediction 2*: In terms of specific resource characteristics, either spatial or temporal resource characteristics might have a stronger influence on the costs of gregariousness. Therefore, either:
*Alternative Prediction 2.1*: Spatial resource distribution (e.g., patchiness and clumpiness) will interact more strongly with gregariousness to influence foraging success,
  or,
*Alternative Prediction 2.2*: Temporal resource variables (e.g., food depletability and renewal) will interact more strongly with gregariousness to influence foraging success.
*Prediction 3*: If social foraging is a bet‐hedging strategy that buffers against energetic shortfall, then greater gregariousness will be associated with lower variance in energy intake over time.

## 2 METHODS

To test the predictions above, we built an agent‐based model in the NetLogo 6.2.2 programming environment (Wilensky, 1999). The model program is available in a public repository at github.com/marcysweber/gregariousness. A full technical model description following the ODD protocol for agent‐based models (Grimm et al., 2006, 2020) is included below (Section 2.1).

### 2.1 Model Summary

**FIGURE 1:**
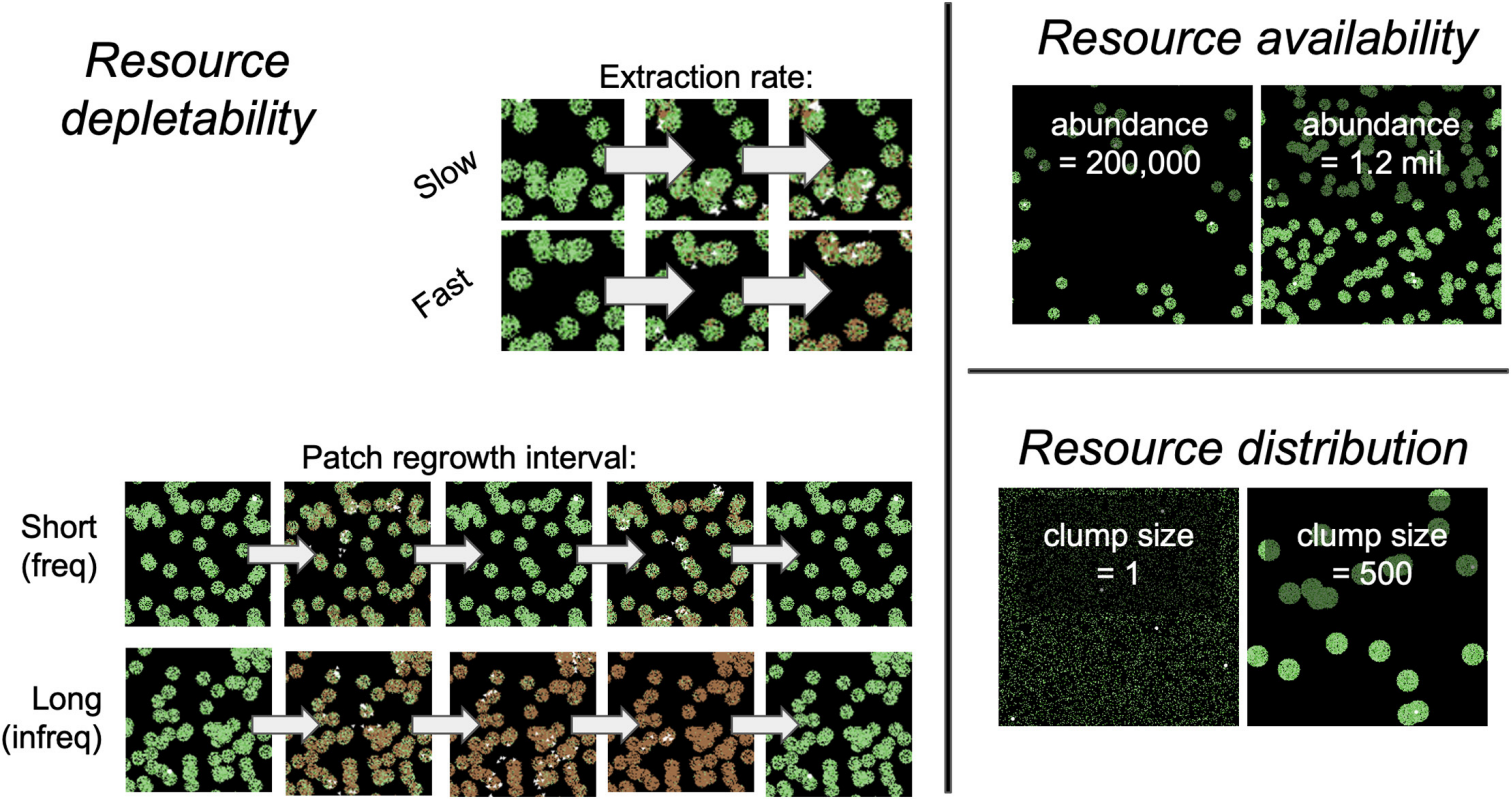
Illustration of the three major categories of food resource characteristics: depletability (left), with parameters extraction rate and patch regrowth interval; availability (upper right), with parameter abundance; and distribution (lower right), with parameter clump size. Green = patches with energy, brown = fully depleted patches, white = primate agents. Depletability parameters are demonstrated over a time series from right to left, with agents consuming resources over time. For resource distribution/clump size, the total amount of food energy is the same on the two panels.

The model contains agents, representing individual primate foragers, and a food resource landscape. Agents are identical at the start of the simulations, except that they are assigned differing competitive strengths (resource holding potential, RHP) which determine which agent wins when they interact aggressively (see Submodels: Deciding to Fight, Fighting, and Xp). The food resource landscape is generated for each simulation instance according to 10 different parameters. These parameters control the amount of energy distributed on the landscape, resource depletability characteristics, and resource distribution characteristics (see Figure 1). For more detail on how the landscape is generated, see Initialization.

**FIGURE 2:**
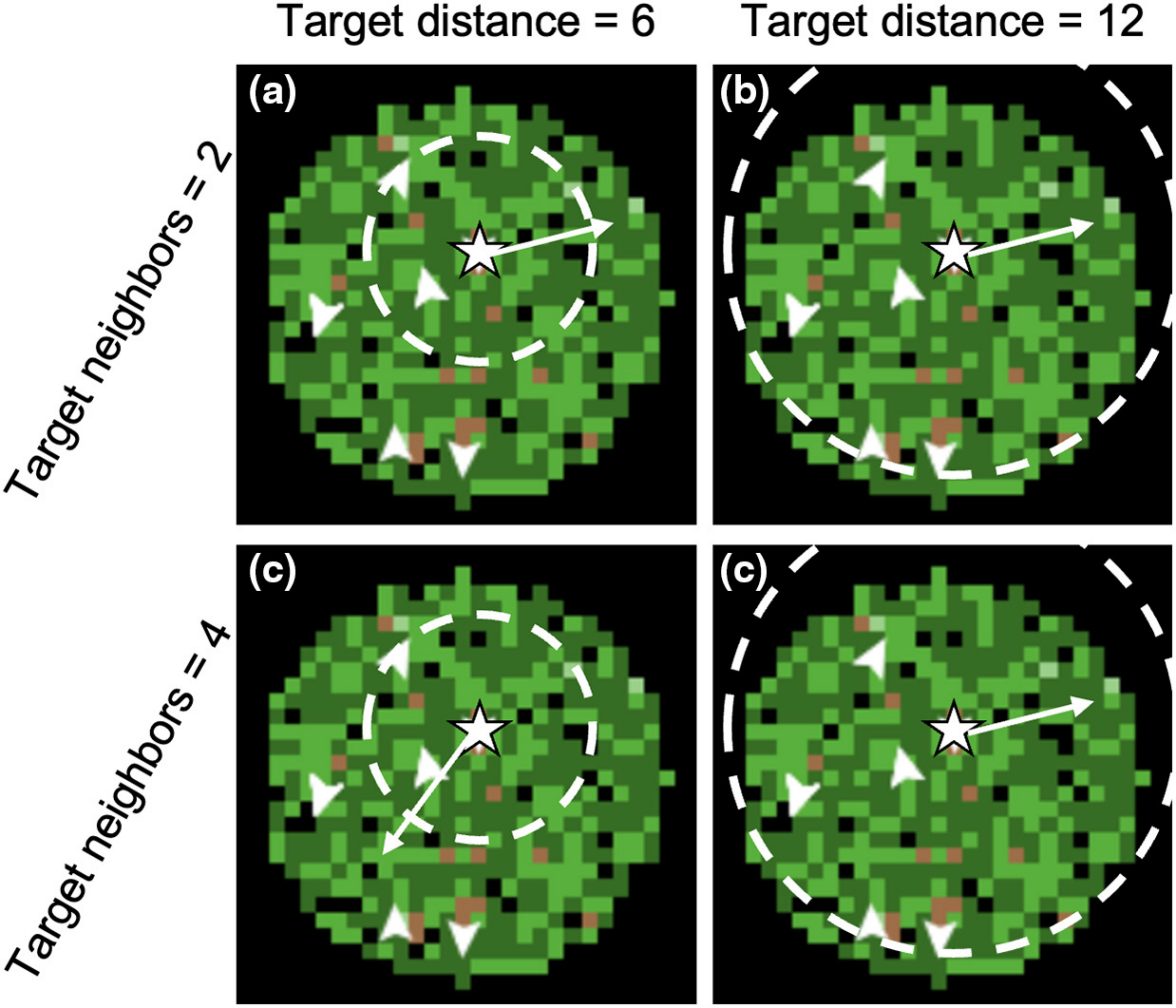
Illustration of the gregariousness rules in the model using the two parameters, target distance and target neighbors. Green and brown squares are resource patches (dark green = highest energy, medium green = moderate energy, and brown = lowest energy); black squares are areas without resources. The white star is the perspective agent; the white arrowheads are the non‐perspective agents. The white dashed line indicates the area with target distance of the perspective agent. The white arrow represents the new heading of the perspective agent. Agents would orient themselves towards the best visible resource patch unless they did not have enough other agents within target distance of themselves. In the left column (a, c), target distance is 6. In the right column (b, d), target distance is 12. In the upper row (a, b), target neighbors is 2. In the lower row (c, d), target neighbors is 4. In a, b, and d, the perspective agent has satisfied gregariousness rules, and will orient towards the highest‐energy resource patch. In c, the gregariousness rules are not satisfied, so the perspective agent will orient towards any visible agents farther away than target distance.

After the agent population and the food resource landscape are generated, agents begin foraging and interacting. As a primary objective, agents seek out the highest‐value food patches. When feeding in a patch, agents can attempt to evict other agents from the patch, with the objective of gaining a greater proportion of the energy left in the patch (see Submodels: Deciding to Fight, Fighting, and Xp, and also see Ekanayake‐Weber et al., 2024). While foraging, agents also have to satisfy certain gregariousness rules which are set by two parameters. Target distance controls the size of a “comfort zone” radius, and target neighbors control the amount of other agents that agents aim to keep nearby. When agents do not have the prescribed number of target neighbors within the target distance, agents will ignore resources and instead turn toward other agents within their sensory range (see Figure 2; see also Submodels: Movement Decisions). Table 1 provides an overview of the model parameters, which control the level of gregariousness and the features of the simulated landscape.

**TABLE 1:** Global model parameters set by sliders in NetLogo's interface tab, and analyzed by Morris Elementary Effect analysis.

Category	Parameter	Description/interpretation	Range of values	MEE?
Gregariousness	tgt‐neighbor	The number of neighbors that each primate must have within the target distance	Integer between 0 and 11	All
	tgt‐distance	The target radius that each primate must maintain a targeted number of primate neighbors within	1–49 patch‐distances	All
Resource characteristics	Abundance	Total amount of energy units to be distributed across the landscape	200,000–1,200,000 energy units	All
	Energy‐per‐capita	Energy units distributed for each primate agent created. Population size calculated by dividing abundance by/ energy‐per‐capita.	4000–9000 energy units	All
	Clump‐size	The mean of the normal distribution which determines the number of patches in a clump	Integer between 0 and 500	All
	Qual‐mean	The mean of a normal distribution which determines energy in patch	Integer between 25 and 150	All
	Qual‐sd	The standard deviation of a normal distribution which determines patch qualities	Integer between 1 and 20	Pattern‐matching only
	Regrowth‐rate	The amount of energy gained by each patch in the landscape at a certain frequency	0.5–1.0 of quality regrown every patch‐regrowth‐freq	Pattern‐matching only
	Patch‐regrowth‐interval	The interval at which patches have the ability to regrow; longer intervals mean less frequent regrowth/more variability over time in resource availability	Integer between 500 and 3000 time‐steps	All
	Extraction‐rate‐mean	The mean of a normal distribution which determines how much energy a primate can extract from a particular patch in single time‐step (i.e., determines depletion speed of patch)	2 to energy units per time‐step	All
	Extraction‐rate‐sd	The standard deviation of a normal distribution which determines how much energy a primate can extract from a particular patch in a single time‐step	0–3 energy units per time‐step	All
Primate movement and sensory characteristics	Movement‐noise	A number that is used to add randomness to a primate's heading when wandering, traveling or dispersing; this is done by subtracting a half of movement‐noise from a random number selected from 0 to movement‐noise and adding to the primate's heading	10–45 degrees	Pattern‐matching only
	Max‐move	The maximum distance that a primate agent can travel in a single time step.	10–50 patch‐distances for pattern‐matching; 25–50 patch‐distances for hypothesis testing	All
	Resource‐detection‐radius	The radius within which a primate can detect all patches with energy units greater than 0	50–100 patch‐distances	Pattern‐matching only
	Other‐primate‐detection‐radius	The radius within which a primate can detect other neighboring primates	50–100 patch‐distances	Pattern‐matching only

### 2.2 ODD protocol model description

#### 2.2.1 Purpose and patterns

The purpose of this model was to investigate the costs and benefits of group living in primates. Specifically, the model was built to explain the impacts of gregariousness (i.e., the behavioral inclination to aggregate with conspecifics) on feeding competition, and how these impacts are mediated by interacting resource characteristics.

The model was calibrated and validated using patterns derived from field data for a wide variety of primate species. Two key patterns of primate foraging behavior, daily path length (Vidal‐Cordasco et al., 2020) and activity budget (Kamilar & Cooper, 2013), were derived from recent comparative studies. Although there are methodological concerns with such data (e.g., see Patterson et al., 2014), here they were only used to decide what range of parameter space to focus on within the model, rather than matching exactly the characteristics of any one species. Daily path length for primates was observed to vary from less than 200 meters per day (*Eulemur fulvus fulvus*), up to extremes of 6 km (*Macaca nigra*), 8.6 km (*Papio hamadryas*), and 10 km (*Homo sapiens*), with 97% of species falling between 0.25 and 4.0 km per day (Vidal‐Cordasco et al., 2020). The ratio of time spent moving versus eating also varied widely, from 0.067 to 0.77 (Kamilar & Cooper, 2013). For approximately 85% of primate species (59 out of 70 species for which activity budget data were available), the ratio of time moving: eating was between 0.25 and 0.6 (Kamilar & Cooper, 2013). Using data from preliminary runs, we calibrated our model to focus on the parameter space which generated daily path lengths between 0.25 and 4.0 km and moving: eating ratios between 0.25 and 0.6 (see Appendix 1 for further details).

#### 2.2.2 Entities, state variables, and scales

The entities of this model were primates and resource patches.

Primates were the agents of the model. The state variables characterizing primates are listed in Table 2. Primate agents did not have any sex or age assigned and did not reproduce.

**TABLE 2:** Variables in the agent‐based model, including agent attributes and patch attributes.

Category	Variable name	Description	States/data structure
Agent attribute	stored‐energy	The amount of energy (in relative units) that the agent has accumulated; all agents start at 30	Real number (float)
	rhp	Resource holding potential: The competitive ability of the agent, assigned randomly at birth/initialization.	Integers 1–8
	nearest‐primates	Primates within visual range (“other‐primate‐detection‐radius”); if more than “tgt‐neighbor”, limited to that number of primates	Turtle‐set of primates
	visible‐resources	Resources tiles currently containing energy within the visual range (resource‐detection‐radius)	Patch‐set
	xp	current value of estimated fighting ability based on previous recorded fight outcomes	Float between 0.0 and 1.0
	exp‐delta‐list	List recording every time the xp was changed by a win or loss	List of floats
	xcor, ycor	Coordinates describing the current location of the agent	Float, float; range, −140 to 140
	heading	The current direction that the agent is pointing; 0 is north, 90 is east, etc.	Degrees out of 360 (clockwise)
Patch attribute	pxcor, pycor	Coordinates describing the location of the patch	Integer, integer; range, −140 to 140
	penergy	The current energetic contents of the patch	Integer; range depends on parameter values
	quality	The maximum energetic contents of the patch, up to which it regrows	Integer; range depends on parameter values
	extraction‐rate	How much energy can be extracted from this patch per tick	Float; range depends on parameter values

Space was represented continuously for primate movement, but was subdivided into discrete patches for representing resources (a patch is a grid cell, not to be confused with clumps of patches with resources). The simulated landscape consisted of a square torus (wrapping horizontally and vertically) of 281 by 281 patches (i.e., 78,961 patches total). Each patch was intended to approximately represent a 10 m × 10 m area of habitat, or, a single tree canopy (as in Chapman et al., 1995). Therefore, the entire landscape represented an area of 2.81 × 2.81 km, or, 7.9 square kilometers. Resource patches were patches containing energy, i.e., feeding sites. All patches were characterized by their coordinates and the variables listed in Table 2. More than one primate could occupy a patch, but patches were “usurpable” (Isbell & Young, 2002), meaning one primate could try to exclude another from the patch they co‐occupied.

For the sake of realism, we varied the agent population size in proportion with resource abundance, but allowed this proportion to vary. Thus, there were two parameters that determined population size: resource abundance (total amount of energy distributed on the landscape) and energy per capita (amount of energy for each primate agent). With resource abundance varying between 200,000 and 1,200,000 energy units, and energy per capita varying between 4000 and 9000 energy units, the population size varied from 22 agents to 300 agents. With the spatial scale mentioned above, this resulted in primate densities ranging between 2.8 individuals per km^2^ to 38.5 individuals per km^2^. See Table 1 for a description of all model parameters.

The model was represented in discrete time steps. Each “tick” (i.e., turn) corresponded to approximately 30 min of daylight time. Thus, 24 ticks represented the events that would take place over the daylight hours of 1 day, assuming equal day and night time hours and ignoring potential effects of seasonal variation in day length. The simulations ran for 4300 timesteps, which represented approximately 6 months.

#### 2.2.3 Process overview and scheduling

Here, we provide an overview; all details of how these processes were implemented are given in the section “Submodels”. Different processes were run at different time scales in the model, i.e., each tick, or, for patch re‐growth, triggered by a parameter.

Within each time step representing half an hour, the following events took place in the following order.

1. Each primate would assess whether the current patch they occupied contained any energy, and then either eat or move.
  - If the current patch, or grid cell, contained energy, the “eat” procedure was called. Before consuming energy from the patch, the primate would sense whether there were other co‐occupants of the current patch, and decide whether to try to displace one of them (“decide_to_fight”; see Submodels). After this decision, if the primate had not been chased out of the patch as a result, they would consume a set amount (“extraction‐rate”) from the patch. Then, their actions were complete for this time step.
  - If the current patch was depleted of energy, the primate would sense other nearby primates (“find_nearest_primates”), and also sense nearby resource patches (“find_visible‐resources”). Then, the “move” procedure was called. The movement involved a combination of maintaining gregariousness rules and moving toward the highest‐energy patch.
  - After all primates had either eaten or moved, each primate had their memory of previous fight wins and losses decay by the appropriate amount, depending on whether their current xp was above or below the running average (see Submodels).

On a longer timescale, resource patches would regrow. The patch regrowth interval was controlled by a parameter, *patch‐regrowth‐interval*, so that it could vary and its influence could be assessed. *Patch‐regrowth‐interval* was the latency to regrow; when patch regrow interval was 500, patches could regrow every 500 timesteps, representing a landscape with renewing and/or seasonally alternating food sources. When patch regrow interval was set to 3000, patches could only regrow every 3000 timesteps. Thus, a higher patch regrowth interval meant that resources remained depleted for longer stretches of time.

#### 2.2.4 Design concepts

##### Basic principles

This model addresses the way in which the costs and benefits of gregariousness are influenced by the characteristics of food resources. Prevailing hypotheses posit that animals which forage socially (such as most anthropoid primates) should experience feeding competition proportionate to the number of other conspecific foragers present, and that this competition is one of the principal costs associated with group living (e.g., Alexander, 1974; Janson, 1988; van Schaik, 1983). Previous agent‐based models (Beauchamp, 2005; Ruxton, 1995) have illustrated that, on simple landscapes, group foragers have lower foraging efficiency, but also lower variance in energy intake. However, feeding competition is known to be heavily influenced by many interacting resource characteristics, including abundance, extraction rate, patch distribution, and patch size (Isbell & Van Vuren, 1996; Koenig & Borries, 2006). Thus, these elements were included in our model. For the sake of simplicity, agents did not behave according to optimal foraging theory (Mangel & Clark, 1986); resource extraction from a patch was constant until a patch was completely depleted, at which point all occupants would move.

##### Emergence

How much energy the primate could gain was the emergent result of their decisions on movement and fight decisions or, with other words, of both direct and indirect competition with other primates, the properties of the landscape, and the gregariousness rules that the primate was obeying. The decision rules were based on implicit assumptions about fitness consequences. The repeated interactions between landscape and behavior allowed travel distances and trajectories to emerge, as well as metrics of foraging efficiency.

##### Adaptation

Agents had three adaptive behaviors. First, if the patch they were currently on did not contain any energy, the agent would decide to leave the patch and attempt to locate a patch with a high amount of energy. Alternatively, while on a patch containing energy, the agent could decide whether to attempt to fight one of the other agents co‐occupying that patch, in order to gain a larger portion of the patch's energy (see Submodel: Deciding to Fight, Fighting, and Xp). Both of these adaptive behaviors sought to maximize energy intake (see Objectives). Finally, an agent would decide to forgo resource foraging in order to maintain proximity to other agents, if its gregariousness rules were not met. This adaptive behavior sought to satisfy (rather than maximize) the objective of the gregariousness rules.

##### Objectives

The primate had the competing objectives of (1) gaining the maximum amount of energy and (2) maintaining a certain distance from a certain number of other primates. The agents' maximizing objective was to accumulate the most energy, which was implicitly linked to real‐world survival, reproductive success, and fitness. Agents expressed this in two ways: moving toward high‐quality resource patches, and attempting to exclude other primates from their current resource patch (see Submodel: Deciding to Fight, Fighting, and xp). By contrast, the gregariousness rules were a satisficing objective, which only altered the behavior of the agents when certain conditions were met (see Submodel: Movement Decisions).

##### Learning

Through their state variable *Xp*, primates kept track and thus learned their own and others' fighting abilities through memory of past conflicts. With more experience, primates were able to make more accurate predictions of who would win or lose future conflicts (see Submodel: Deciding to Fight, Fighting, and Xp). Agents did not learn or remember the locations of resource patches or clumps.

##### Prediction

Agents attempted to accurately predict the outcome of a possible fight before deciding to attack another agent. If the agent predicted that they would lose, they were less likely to decide to attack. This prediction was done using the attribute “Xp” (see Submodel: Deciding to Fight, Fighting, and Xp).

##### Sensing

Primates sensed information about the environment, themselves, and other primates.

Primates sensed the location of resource patches within the zone of *resource‐detection‐patch‐radius*, and they could sense the relative current energy contents of these patches. Primates could also sense the current location of other nearby primates within other‐primate‐detection‐radius, up to the count of the current tgt‐neighbor value (see Table 2 and Submodels). Primates could also sense the *Xp* of themselves and primates on the same patch as themselves. *Xp* was reflective of a “memory” of past aggressive encounters, and were used to estimate the costs of engaging in a future encounter (see Submodels: Deciding to Fight, Fighting, and Xp).

##### Interaction

Primate interacted both indirectly and directly in this model. Primates interacted indirectly by consuming resources. If the patch's energy was greater than zero, then the primate could consume an amount determined by the patch's extraction‐rate. This energy was then unavailable to other primates until the patch could regrow.

Primates directly interacted according to their gregariousness rules. When they did not have the “target neighbors” amount of other agents within the radius of the target distance, then the agent would move toward the average location of the primates that were visible to them (within other‐primate‐detection‐radius). Primates also directly interacted by fighting to try and evict each other out of a shared patch (see Submodels: Deciding to Fight, Fighting, and Xp).

##### Stochasticity

Processes driven by pseudorandom numbers were the following: At initialization, the assignment of each patch's energy contents was drawn from a normal distribution defined by the parameters qual‐mean and qual‐sd (Table 1), and the assignment of each patch's extraction (a measure of the speed by which agents could gain energy from the patch) rate was drawn from a normal distribution controlled by the parameters extraction‐rate‐mean and extraction‐rate‐sd. During the movement procedure “wander” (and “travel” when target neighbors was 0), the agent made small turns right or left according to a randomly generated number between 0 and the parameter “movement‐noise.” The decision to attack another agent was also determined by a randomly generated value between 0.0 and 1.0 (see Submodels: Deciding to Fight, Fighting, and Xp).

##### Collectives

While following gregariousness rules, agents would form emergent spatial aggregations of agents which could be considered as collectives, but these collectives were not entities by themselves, as they had no own behavior. Clumps were collectives of resource patches, whose size determined the overall resource distribution of the simulated landscapes.

##### Observation

The model's outcomes were observed through a few summary variables. For hypothesis 1, we observed energy intake rate (energy gained divided by ticks, averaged across the population), and daily distance traveled (distance traveled divided by (ticks divided by 24)), averaged across the population. For hypothesis 2, we observed monthly variance in energy intake, and biweekly variance in energy intake, but only further analyzed monthly variance.

#### 2.2.5 Initialization

The “setup” procedure initialized each unique simulation of the model. The starting conditions of each simulation were controlled by a number of resource parameters (Table 2, Figure 1). A resource landscape of 281 × 281 patches was created, according to the parameter settings on the Graphical User Interface, a BehaviorSpace experiment (a NetLogo tool to define simulation experiments; Wilensky & Shargel, 2001), or a simulation experiment defined and run from R using the nlrx package (Salecker et al., 2019). The setup procedure would create new clumps of resource patches (using the parameter “clump‐size”) and fill in resource patches in these clumps (using the parameters “qual‐mean” and “qual‐sd”) until the total desired amount of energy was distributed on the landscape (as prescribed by the parameter “abundance”). Clumps could overlap, in which case very dense “super‐clumps” would form, and any “leftover” energy from a clump whose area already had many patches filled in would simply be distributed in another new clump. The primate population size was calculated by dividing the resource abundance by the energy‐per‐capita. Then, that amount of primate agents were produced, each assigned a resource holding potential, *rhp* (determining competitive strength), between 1 and 8, and placed on one of 5 randomly selected resource patches from across the entire landscape. Primates began on this limited number of spawn points so that they did not have to search randomly for resources at the very beginning of the simulation, nor search for other agents, which could have artificially inflated the variance in distance traveled and foraging efficiency.

#### 2.2.6 Input data

The model does not use input data to represent time‐varying processes.

#### 2.2.7 Submodels

##### Movement decisions

Agents would move when the patch they were occupying no longer contained any energy. Agents did not pay any explicit cost for movement, in terms of energy. However, we focused on foraging efficiency (energy intake divided by distance traveled) as our main foraging metric, since this incorporates both benefits and costs. Agents only responded to resources and other agents within their sensory range; they had no memory of the locations of previously visited resources. In a single time‐step, agents could move as many patch distances as the parameter “max‐move” indicated. Agents could also take steps that were shorter than max‐move, if the selected destination patch was less than max‐move patch distance away. Before taking a “step”, agents would decide how to adjust their heading. This was a combination of being attracted to the highest‐value patches and obeying gregariousness rules (see Figure 2).

*Wander and travel*. If there were no resource patches within the sensory range of the agent (resource‐detection‐radius), and there were no other agents detected nearby *or* target neighbors = 0, the agent would have their heading changed a random amount as limited by the parameter movement‐noise (corresponding to a correlated random walk; procedures “wander”/“travel” when target neighbors = 0). If there were no resource patches within sensory range but there were other agents nearby *and* target neighbors was ≥1, then the agent would change their heading to be the average direction toward all visible conspecifics (procedure “travel”).

*Forage*. The agent had satisfied their gregariousness rules if they had the appropriate number of neighbors (set by target neighbors) within the appropriate distance (set by target distance; see Figure 2a,b,d). If both conditions were met, the agent would adjust their heading toward the patch with the most energy within their sensory range (procedure “forage”). While moving for multiple timesteps in a row, agents would maintain the same goal patch (“patch‐picked”) rather than picking a new destination each timestep, so long as the patch‐picked was still within sensory range.

*Cohere*. If target neighbors ≥1 and the agent did not have enough target neighbors within the target distance, then the agent would ignore resources in favor of attempting to fulfill its gregariousness rules (procedure “cohere”; see Figure 2c). If there were other agents within sensory range but outside the target distance, the agent would adjust their heading to be directed toward the average location of visible other agents. If there were no other agents sensed outside of the target distance, the agent would revert to foraging (see above) for that timestep. If target neighbors = 0, the agent would only wander and forage.

##### Deciding to fight, fighting, and Xp

If the patch that an agent was occupying at the beginning of their turn contained energy, then the agent had the option to try to fight one other agent occupying the same patch (see “Schedule”). As an outcome of a fight, the loser was forced to “flee” the patch. Therefore, it was in each agent's best interest to evict weaker agents out of a patch to avoid having to share food, but to not start fights which they would lose, since this would deprive themselves of the resource. Agents made this choice using a procedure called “decide_to_fight,” which was greatly influenced by the design of previous models including DomWorld (Hemelrijk, 1999, 2000) and the earlier MIRROR modeling methodology, developed for bee behavior (Hogeweg & Hesper, 1983, 1985). Detailed testing of this aspect of the model is described elsewhere (Ekanayake‐Weber et al., 2024). Upon selecting an opponent randomly from the other patch occupants, the agent would estimate benefits of fighting (how much more energy they were likely to gain with the opponent evicted) and the costs of fighting (comparing Xp, based on the history of previous fights; see below). The agents determined the benefits (*B*) of attacking by comparing the energy gains they could make by evicting another primate from the current patch to the availability of energy in neighboring patches (Vogel & Janson, 2011). This was calculated as follows:

Benefits calculation.

(1) $$B=\frac{\frac{{\text{penergy}}_{c}}{\text{count}}-\frac{{\text{penergy}}_{c}}{\text{count}-1}}{\text{mean}\left({\text{penergy}}_{a}\right)}$$

where penergy_c_ is the current energy contents of the current patch, count is the total number of agents currently located on that patch, including the self, and penergy_a_ is the current energy contents of each of the patches neighboring the current patch. In other words, the benefit of attacking is the difference between the current portion of energy the agent can expect and the portion of energy they could expect with one fewer competitor on the patch, relative to what they might gain by moving to a neighboring patch (assuming they have no knowledge of what competitors might be on those neighboring patches). Therefore, when most of the nearby energy was contained in the current patch, agents were more likely to attack co‐occupants; when neighboring patches had similar or higher penergy, agents were less likely to attack.

Costs were estimated to be high if the opposing agent was particularly strong (high xp) and/or the approaching agent was weak (low xp); conversely, costs could be estimated to be low if the approaching agent appeared strong and/or the opposing agent appeared weak. costs of attacking were estimated as follows:

Costs estimation for experience‐based decision‐making.

(2) $$C=\frac{{\mathrm{xp}}_{\mathrm{opp}}-{\mathrm{xp}}_{\text{self}}+1}{2}$$

The formula was designed to vary between 0.0 and 1.0. When opponents were equal (xp_opp_ − xp_self_ = 0), the formula was designed to return a cost estimation of 0.5.

To compare the benefits and cost estimations, costs (*C*) and benefits (*B*) were weighed equally, as both were put on a 0.0 to 1.0 scale. The decision to attack was stochastic, such that the likelihood of attack was

Cost–benefit analysis in the decision to attack.

(3) $$P\left(\text{attack}\right)=\frac{B}{B+C}$$

Therefore, as the benefits increased relative to the costs, the approaching agent had a higher probability of attacking, and as the costs increased relative to the benefits, the approaching agent had a decreasing probability of attacking. When costs and benefits were equal (whether low or high), the chance of attacking was 0.5.

If the agent decided to fight the opponent (as described above), then the winner was determined based on the two agents' resource holding potentials (RHPs). RHP represented the overall fighting ability of the agent, which in many species is a combination of body size, body weight, agility, fighting experience, and weaponry, among other factors (see table 1 in Arnott & Elwood, 2009). RHP was assigned randomly to all agents at the beginning of each simulation, and had a value between 1 and 8 which did not change during the simulations. The winner of the fight was always the agent with the higher RHP. If two agents had the exact same RHP, then the aggressor would lose the fight.

After each fight, the combatants “remembered” whether they had won or lost, using the variable “xp.” Xp was an attribute that each agent possessed, always beginning at 0.5 and ranging from 0 to 1.0. When an agent won a fight, xp was increased by 0.01 (unless xp was already above 0.98). When an agent lost a fight, xp was decreased by 0.01 (unless xp was already below 0.02). Thus, repeated losses over time would lead to xp << 0.5 and repeated wins would lead to xp >> 0.5. It was in this way that agents assessed each other's competitive abilities, relative to their own. Xp also decayed over time toward a running average of the previous five fight outcomes.

### 2.3 Analysis

**FIGURE A1:**
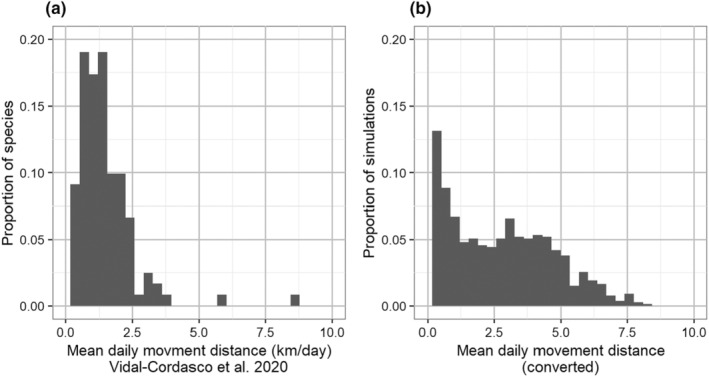
Daily path length comparison between data set from Vidal‐Cordasco et al. (2020) (a), and model output (b). In the agent‐based model, each patch‐distance was representative of 10 m.

**FIGURE A2:**
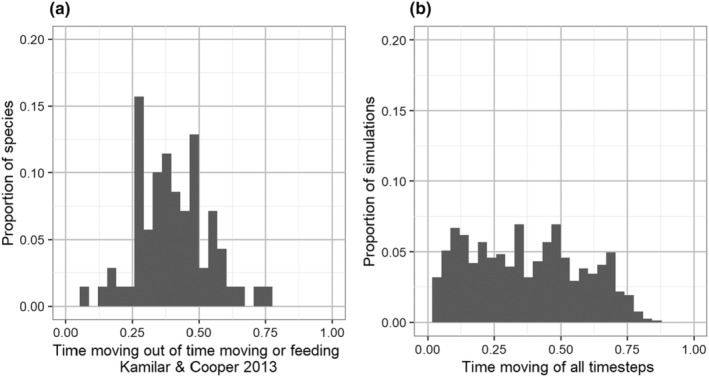
Activity budget comparison between data set from Kamilar and Cooper (2013) (a), and model output (b). Activity budget was simplified to a ratio of time moving to time eating, because the design of the agent‐based model did not include resting or grooming time.

**FIGURE A3:**
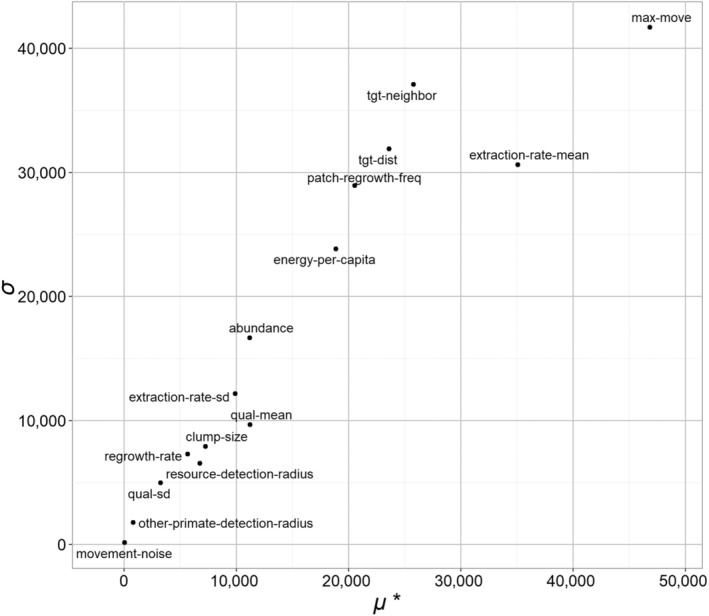
Morris Elementary Effects for the mean distance traveled, with all possible parameters varying.

**FIGURE A4:**
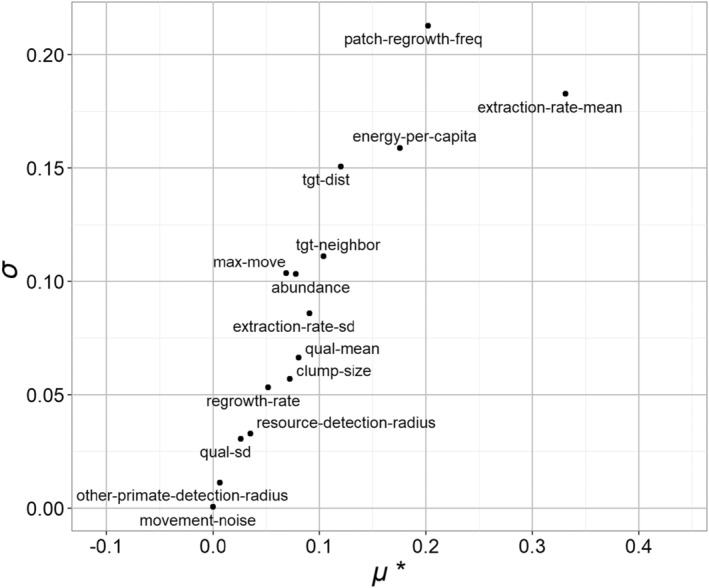
Morris Elementary Effects for the proportion of time that agents spent moving, relative to eating, with all possible parameters varying. Note that the same 5 parameters as in Figure OS2.3 have the lowest *σ* and μ* values, which led to their exclusion from further analysis.

Before proceeding with hypothesis tests, we verified that the model conformed to patterns observed in species of interest (Gallagher et al., 2021; Grimm et al., 2005). We compared the range of daily movement distances generated by our resource configurations to the ranges observed across the primate order (Vidal‐Cordasco et al., 2020). We performed Morris Elementary Effects (MEE) sensitivity analysis (Morris, 1991; Saltelli et al., 2008) using the nlrx package (Salecker et al., 2019) in R (R Development Core Team, 2016) for the purposes of pattern‐matching and for testing hypotheses. This technique enabled us to vary all 15 parameters initially, including those background parameters which were not of interest to the main study, but which nonetheless might have an impact on the results (see Table 1, Appendix 1 for details). Using MEE analysis also allowed us to efficiently and accurately identify which parameters were most contributing to anomalous results during preliminary runs, and to gradually adjust these parameter ranges until we had sufficient pattern matches with daily travel distance and activity budget (see Figures A1 and A2). The MEE analyses for pattern matching included all of the parameters listed in Table 1. This parameter space utilized initially is referred to below as the general scenario. From all these MEE results, we took the elementary effects μ*, a measurement of main effects, and *σ*, a measurement of interaction and non‐linear effects, and used them as coordinates to plot the parameters (Morris, 1991; Saltelli et al., 2008). From these plots (Figures A3 and A4), we could visually determine which parameters had small effects and which had large effects.

When testing our predictions, the parameters which were determined by the MEE to have large effects underwent further analysis. First, the raw data produced by the MEE were visualized as heatmaps, which made many of the main and interaction effects salient. To clarify the strongest effects, more detailed heatmaps were produced (e.g., Figure 4) with all permutations of parameters of interest run for 10 replicates, and all background parameters held constant (see Table A1). After performing all of the above, we constructed the resource configurations that we predicted would create the strongest and weakest effects of gregariousness, which were a fast‐extracting, slow‐renewing scenario and a slow‐extraction, fast‐renewing scenario respectively.

**FIGURE A5:**
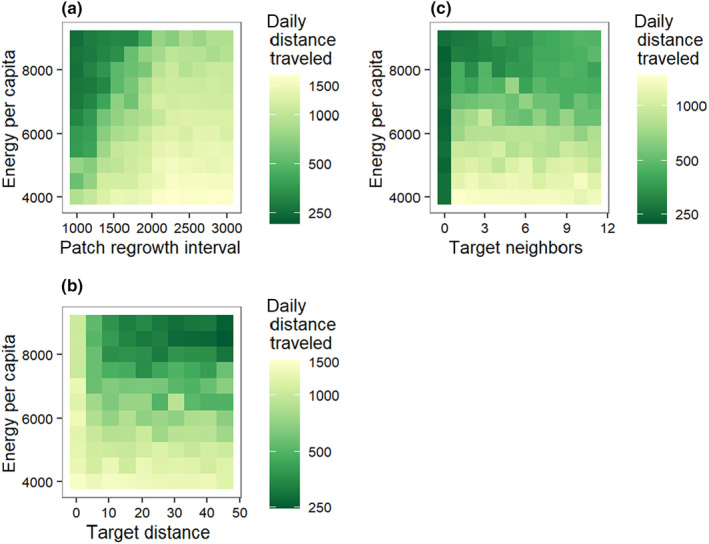
Additional heatmaps not included in Figure 4. Based on the Morris Elementary Effect results (see Figure 3), pairwise experiments were designed for all permutations of significant parameters. The above heatmaps represent the interactions of (a) energy per capita and patch regrowth interval, (b) energy per capita and target distance, and (c) energy per capita and target neighbors. These were excluded from Figure 4 on the basis that the effects of energy per capita are exactly as expected; as energy becomes more scarce and population density goes up (lower energy per capita), agents travel farther distances per day.

For predictions 1 and 2, we measured both population mean energy intake rate (energy units gained divided by the number of time steps, 4300) at the end of the simulation run, and the population mean daily distance traveled (total patch‐length traversed divided by the number of model days) at the end of the simulation run. For prediction 3, we measured mean variance in individual energy intake at a 720 time‐step interval (i.e., approximately monthly). This scale of variance should be biologically relevant from the perspective of avoiding starvation (Figure A5).

## 3 RESULTS

**FIGURE 3:**
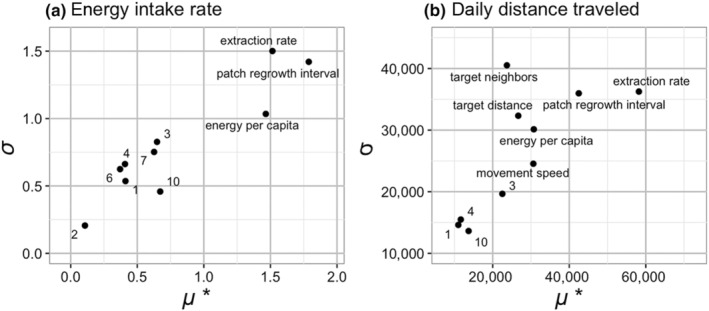
Results of a Morris Elementary Effects sensitivity analysis for energy intake rate (a) and daily distance traveled (b) in a general scenario. μ*, on the *x*‐axis, is a measurement of the main effect of each parameter. *σ*, on the *y*‐axis, is a measurement of the interaction and non‐linear effects of each parameter. For each plot, the scale of the *x* and *y*‐axes are in values of that outcome, that is, energy units gained per unit time for (a), and meters traveled per day for (b). The most influential parameters are clustered in the upper right corner, while the least influential cluster towards the lower left corner. Numbered data points refer to the following parameters, in order from smallest to largest main effects: 1, absolute resource abundance; 2, maximum movement distance; 3, extraction rate standard deviation; 4, clump size; 6, target neighbors; 7, target distance; 10, mean patch quality.

A Morris Elementary Effects (MEE) experiment ran 792 simulations of varying parameter configurations in a general scenario, designed to capture the range of parameter space relevant to living primates (see Appendix 1). The MEE analysis showed that three parameters had a large effect on energy intake rate: patch regrowth interval, mean extraction rate, and energy per capita (Figure 3a). All other parameters had very little main or interaction effects on energy intake, including the gregariousness parameters. Five parameters had a large effect on daily distance traveled: mean extraction rate, patch regrowth interval, energy per capita, target distance, and target neighbors (Figure 3b). The parameter for movement speed, *max‐move*, also had a moderate main effect on daily distance traveled, but this is to be expected, since movement speed will limit the distance agents are able to travel. Since daily distance traveled showed a much stronger effect of our gregariousness parameters than energy intake rate did, we chose to further analyze daily distance traveled.

**FIGURE 4:**
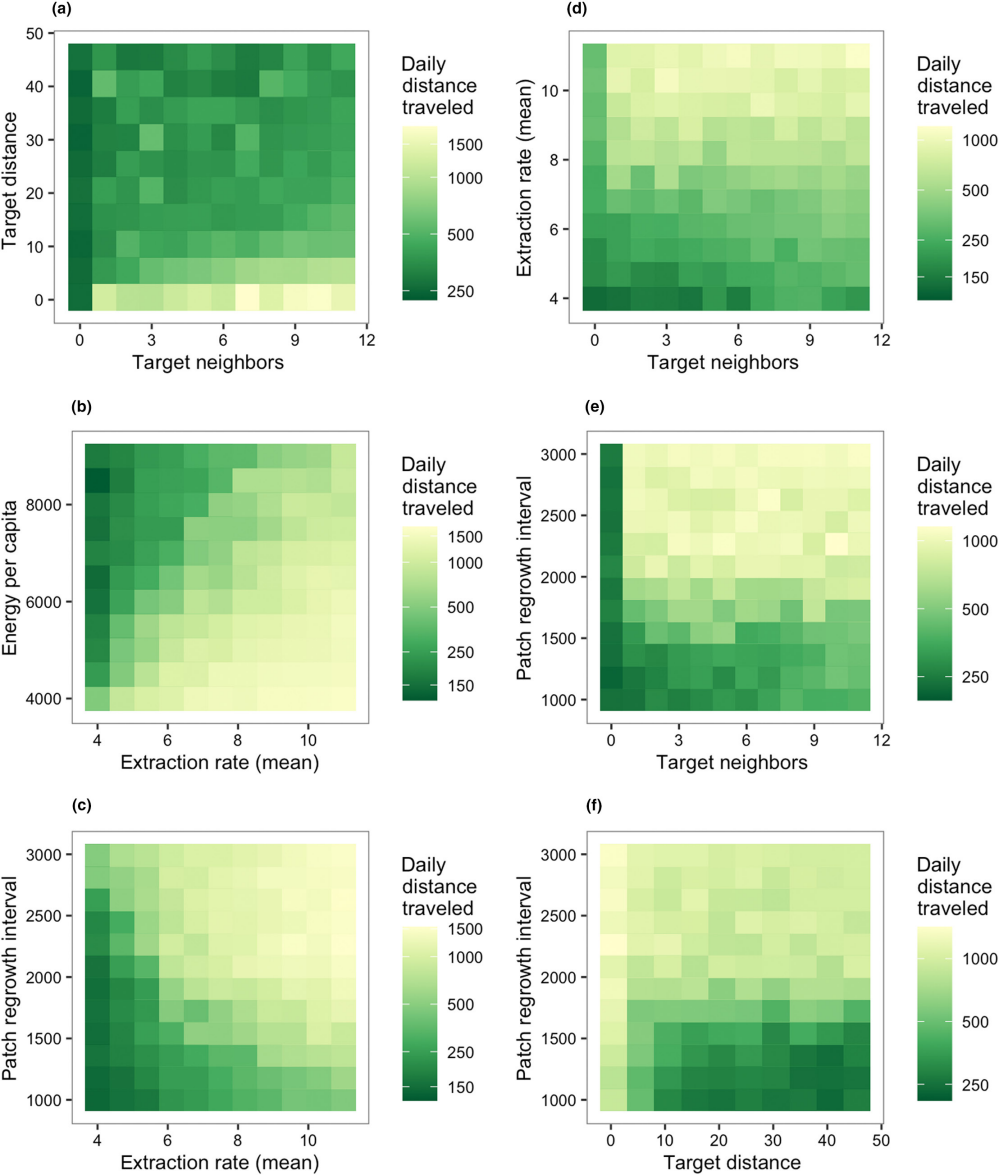
Resource effects in the general scenario. Darker color indicates longer distance traveled per day. Across the entire parameter space representative of primates, foraging efficiency was highest when extraction rate was slower, patch regrowth interval was shorter, and patches contained more energy. (a), Interaction between the two gregariousness parameters, target neighbors and target distance. (b) Interaction between energy per capita and extraction rate (mean). (c) Interaction between extraction rate (mean) and patch regrowth interval, the two most influential parameters. (d) Interaction between target neighbors and extraction rate (mean). (e) Interaction between target neighbors and patch regrowth interval. (f) Interaction between target distance and patch regrowth interval.

Based on the results of the MEE, we examined the five most influential parameters on daily distance traveled in more detail. First, we considered how the two gregariousness parameters, target neighbors and target distance, impacted daily distance traveled. There was an interaction between target neighbors and target distance, whereby having lower target neighbors and/or higher target distance, i.e., a low local forager density, caused the agents to travel shorter distances each day (Figure 4a). Only if target neighbors was zero (i.e., solitary foraging) was daily distance traveled always short, regardless of target distance.

Next, we examined how the resource characteristics affected feeding competition (Figure 4b,c and Figure A5a). First, there was a straightforward effect of energy per capita, such that when energy per capita was lower (i.e., population density relative to calories is higher), there were longer daily travel distances. This was most apparent when observing the combined effects of energy per capita and mean extraction rate (Figure 4b). Patch regrowth interval also interacted strongly with mean extraction rate (Figure 4c). Slower extraction rates and shorter patch regrowth intervals (i.e., more frequent renewal) were associated with shorter daily distances traveled, whereas faster extraction and longer patch regrowth intervals (i.e., less frequent renewal) were associated with longer daily distances traveled.

Then, we examined how the gregariousness parameters interacted with the resource characteristics (Figure 4d–f and Figure A5b,c). Mean extraction rate interacted with target neighbors, such that high target neighbors was associated with longer daily distances traveled even at slower extraction rates, but when target neighbors was zero, daily distances traveled were relatively short even at the fastest extraction rates (Figure 4d). Similarly, patch regrowth interval interacted strongly with both gregariousness parameters. Although patch regrowth interval greatly affected daily distance traveled overall, this effect was overridden by having a target neighbors of zero (which consistently was associated with short daily distances traveled; Figure 4e) or having a very short target distance (which was consistently associated with long daily distances traveled; Figure 4f).

**FIGURE 5:**
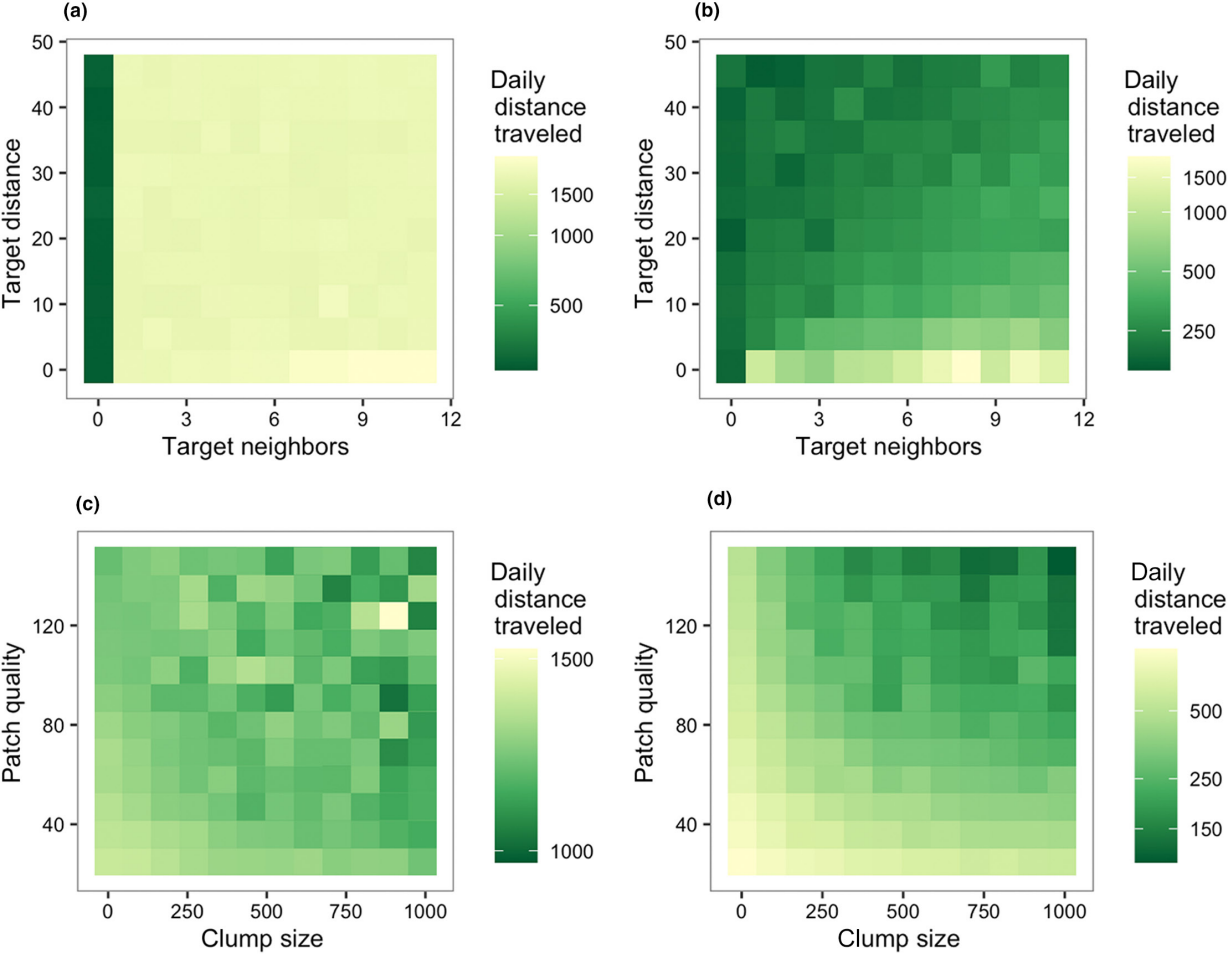
Comparison of outcomes of the fast‐extracting, slow‐renewing scenario (a, b) and the slowextracting, fast‐renewing scenario (c, d). Darker color indicates shorter average distance traveled per day. (a) Non‐interaction between the two gregariousness parameters in the fast‐extracting, slow‐renewing scenario. (b) Inconsistent interaction between patch quality and clump size in the fast‐extracting, slowrenewing scenario. (c) Interaction between the gregariousness parameters in the slow‐extracting, fastrenewing scenario. (d) Interaction between patch quality and clump size in the slow‐extracting, fastrenewing scenario.

**FIGURE A6:**
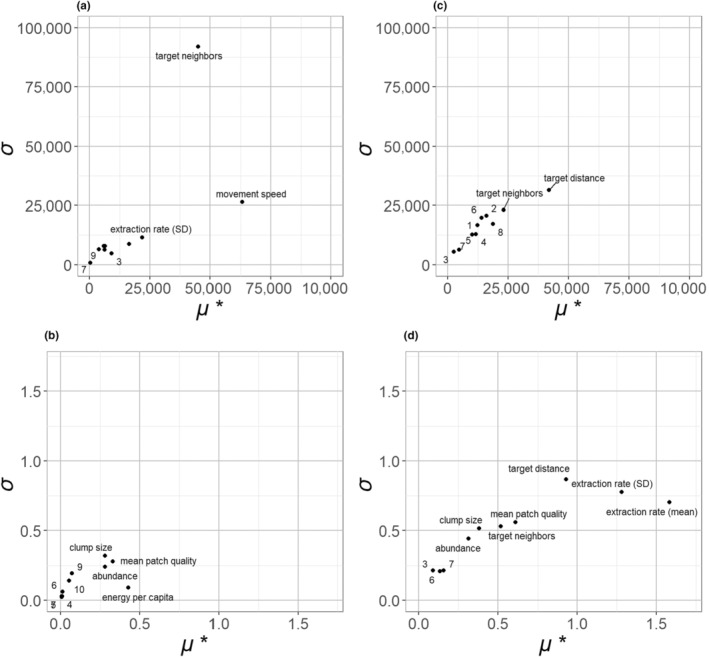
Morris Elementary Effects for the fast‐extracting, slow‐renewing scenario (a, daily distance traveled; b, energy intake) and the slow‐extracting, fast‐renewing scenario (c, daily distance traveled; d, energy intake). Scales for each outcome have been made equal, for easier comparison. Numbered data points are, 1 = abundance; 2 = clump size; 3 = energy per capita; 4 = extraction rate mean; 5 = extraction rate standard deviation; 6 = movement speed; 7 = patch regrowth interval; 8 = mean patch quality; 9 = target distance; 10 = target neighbors.

Using the results above, we constructed two additional scenarios to focus on contrasting regions of the parameter space. In one scenario, we focused on a fast‐extracting, slow‐renewing region of parameter space, designed to exaggerate the costs of gregariousness through high depletability. In another scenario, we focused on a slow‐extracting, fast‐renewing region of parameter space designed to weaken the costs of gregariousness through low depletability. The two scenarios differed greatly in outcome (see Figure A6 for full MEE results). In the fast‐extracting, slow‐renewing scenario, target neighbors had by far the largest impact on daily distances traveled, because only with a target neighbors of zero (non‐gregariousness) was daily distance traveled short (Figure 5a). There were also some secondary effects of parameters for clump size and patch quality, but these were inconsistent (Figure 5b). By contrast, in the slow‐extracting, fast‐renewing scenario we observed a subtle effect of both gregariousness parameters, as well as a clearer interaction effect between patch quality and clump size (Figure 5c,d). Daily distance traveled was longer when target distance was short and target neighbors was high (i.e., high forager density), but daily distance traveled was shorter with longer target distance and/or fewer target neighbors (Figure 5c). In the slow‐extracting, fast‐renewing scenario, daily distance traveled was also impacted by spatial resource distribution: daily distance traveled was shorter when patch quality was higher and clump size was larger (Figure 5c).

**FIGURE 6:**
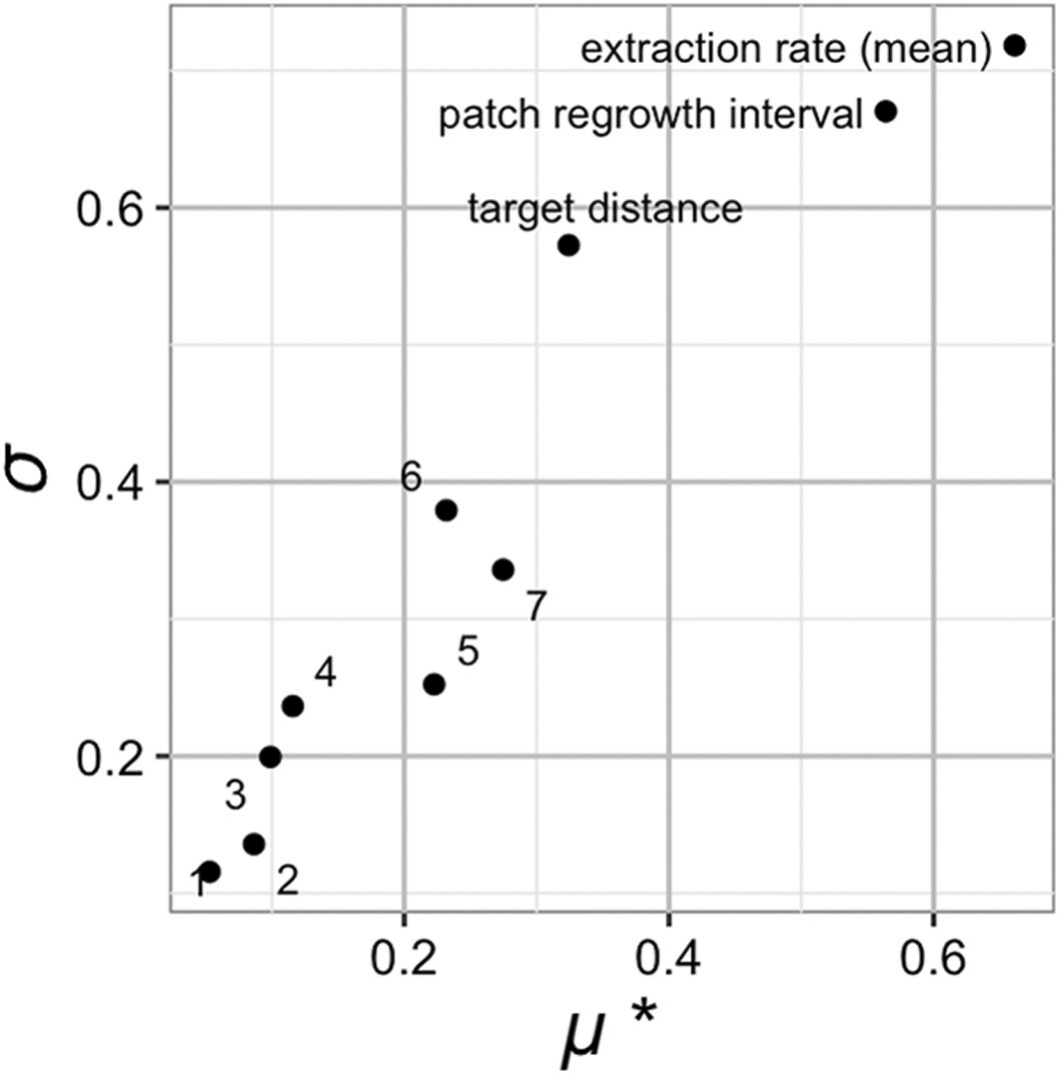
MEE of month‐to‐month variance in energy intake in the general scenario. μ*, on the *x*‐axis, is a measurement of the main effect of each parameter. *σ*, on the *y*‐axis, is a measurement of the interaction and non‐linear effects of each parameter. The most influential parameters are clustered in the upper right corner, while the least influential cluster towards the lower left corner. Numbered data points refer to the following parameters, in order from smallest to largest main effects: 1, maximum movement speed; 2, total resource abundance; 3, target neighbors; 4, clump size; 5, mean patch quality; 6, energy per capita; 7, extraction rate standard deviation.

Finally, we examined how variance in energy intake was influenced by resource parameters and gregariousness. Morris Elementary Effects analysis revealed that only three parameters had a large effect on variance in energy intake: mean extraction rate, frequency of patch regrowth, and target distance (Figure 6). All other parameters were much less influential.

**FIGURE 7:**
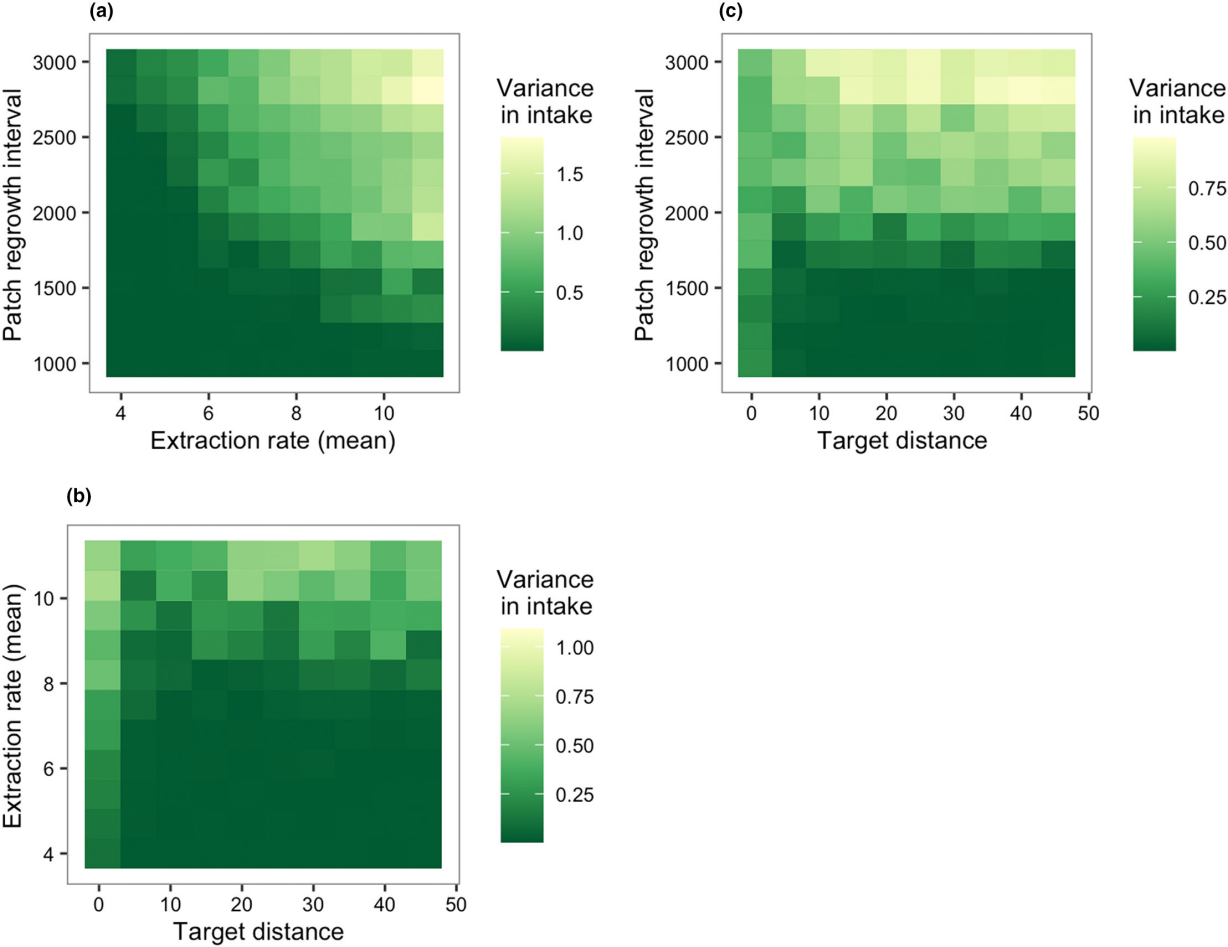
Interaction effects on month‐to‐month variance in energy intake in the general scenario. (a) Variance in intake increases with faster extraction rate and longer patch regrowth interval. However, under these conditions, shorter target distance can reduce variance. In (b), at the highest levels of extraction rate (>10), variance is lower when target distance is between 5 and 15 than when target distance is between 20 and 30. For lower values of extraction rate, variance was highest when target distance = 0. Similarly, in (c), when the patch regrowth intervals were the longest (>2500), having a target distance of 10 or lower led to lower variance in intake than when target distance was longer. For shorter patch regrowth intervals, target distance = 0 had similar or higher variance than other values of target distance.

**FIGURE 8:**
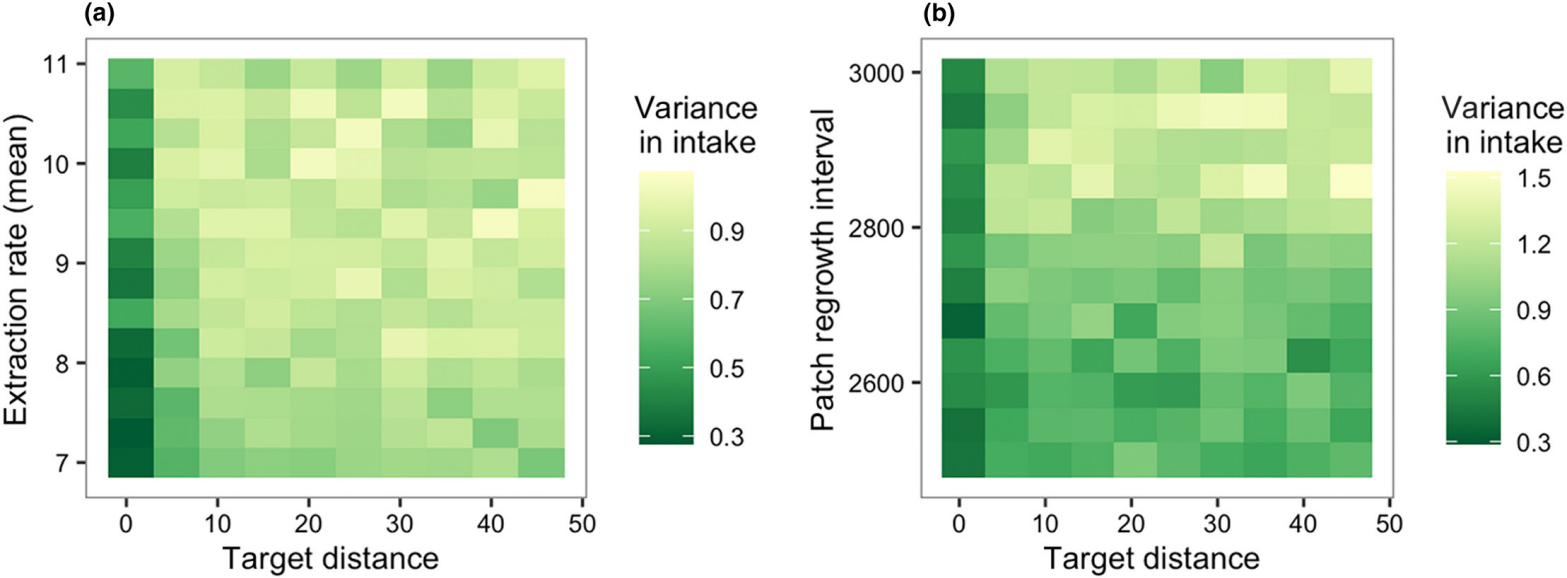
Interaction effect on variance in intake in the fast‐extracting, slow‐renewing scenarios. Target distance still provided a protective effect against too much variance in intake. (a) The interaction effect of target distance and extraction rate, when patch regrowth interval was also long. Variance in intake was consistently lower when target distance = 0. (b) The interaction effect of target distance and patch regrowth interval, when extraction rate was also fast. Variance in intake was frequently lower when target distance = 0, especially at the longest patch regrowth intervals.

**FIGURE 9:**
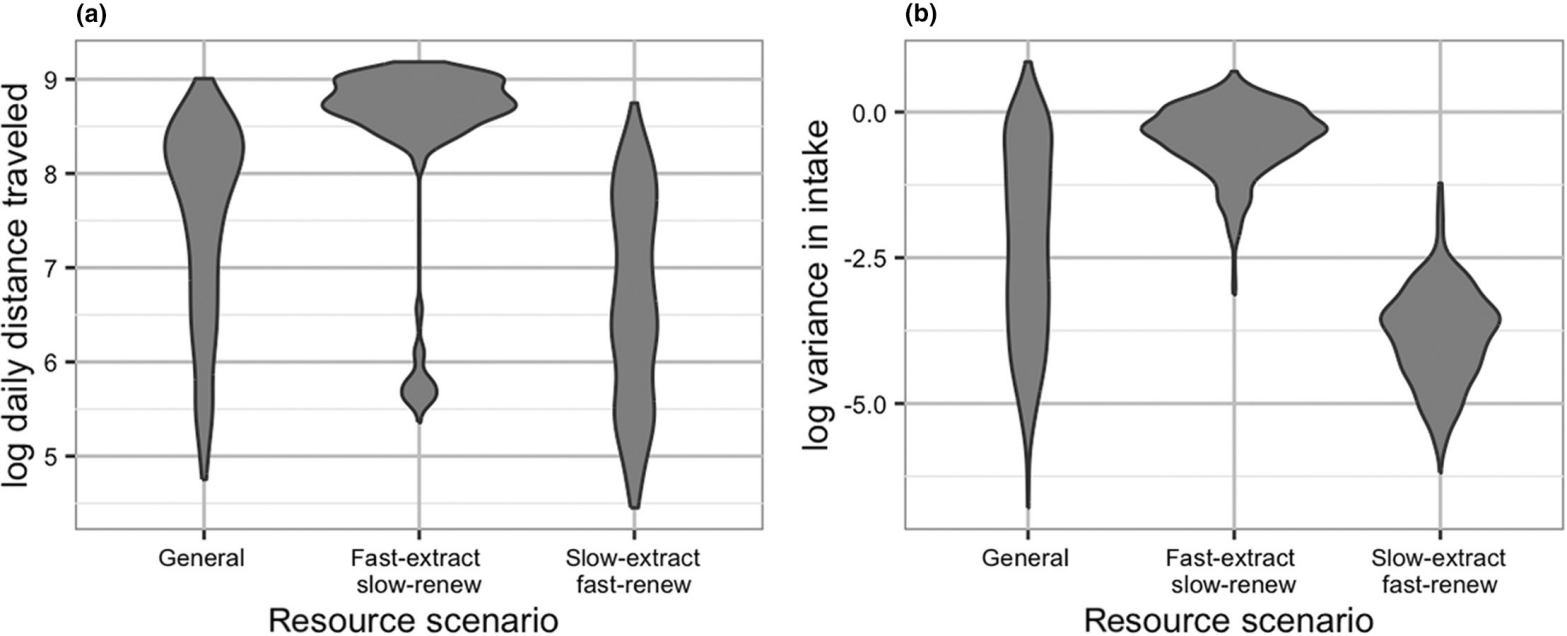
Comparison of both daily distance traveled (a) and variance in intake (b) for the three scenarios that compared regions of parameter space: general, fast‐extracting/slow‐renewing, and slow‐extracting/fast‐renewing. Both daily distance traveled and variance in energy intake have been logtransformed for the purposes of visualization. Overall, the fast‐extracting, slow‐renewing scenario created longer daily distances traveled and higher variance in intake, while the slow‐extracting, fast‐renewing scenario created slightly shorter daily distances traveled and much lower variance in intake.

**FIGURE A7:**
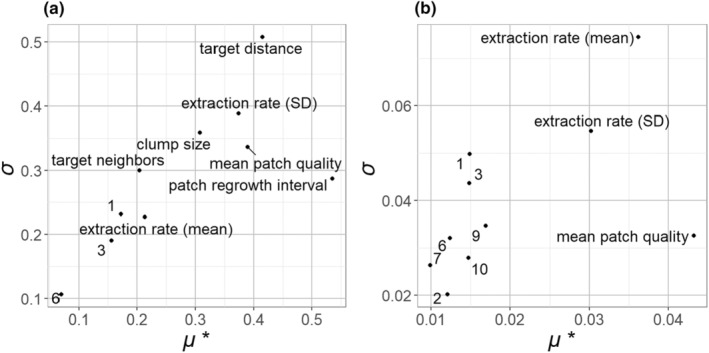
Morris Elementary Effects for variance in energy intake in the fast‐extracting, slow‐renewing scenario (a) and the slow‐extracting, fast‐renewing scenario (b). Note that the scale of the axes in (b) are approximately one‐tenth that of the scale of (a), indicating that the overall effects in the slow‐extracting, fast‐renewing scenario were of a much smaller magnitude than those in the fast‐extracting, slow‐renewing scenario. Numbered data points are, from lowest influence to highest: 1 = abundance; 2 = clump size; 3 = energy per capita; 4 = extraction rate mean; 5 = extraction rate standard deviation; 6 = movement speed; 7 = patch regrowth interval; 8 = mean patch quality; 9 = target distance; 10 = target neighbors.

Again, we examined the most influential parameters identified by the MEE in more detail. There were clear interactions between all three influential parameters (Figure 7). A combination of high mean extraction rate and long patch regrowth interval was associated with higher variance in energy intake (Figure 7a). However, shorter target distances seemed to reduce variance in intake at the highest extraction rates and longest patch regrowth intervals (Figure 7b,c). We also investigated how variance in energy intake was impacted by the fast‐extracting, slow‐renewing and slow‐extracting, fast‐renewing scenarios (Figures 8 and 9). Target distance had a significant impact on variance in energy intake in the fast‐extracting, slow‐renewing scenario (see Figure A7a), where short target distance was associated with lower variance in intake (Figure 8a,b). In the slow‐extracting, fast‐renewing scenario, gregariousness did not have any effect on variance in energy intake (see Figure A7b), and the overall variance in intake was much lower in this scenario than in the fast‐extracting, slow‐renewing scenario (Figure 9b).

## 4 DISCUSSION

The foraging costs and benefits of gregariousness have remained difficult to analyze (Beauchamp, 2005; Georgiou et al., 2022; Ruxton, 1995). Here, we designed an agent‐based model to disentangle the effects of gregariousness and resource characteristics on foraging. With this model, we sought to answer two questions: (1) how do gregarious behaviors interact with particular resource characteristics to affect foraging? and (2) in what circumstances might gregariousness have very low costs, or even a feeding benefit?

As expected, we found that indirect effects of feeding competition (specifically, daily distance traveled) were influenced by a combination of food resource characteristics and gregarious behavior. Food extraction rate and patch regrowth interval were the most influential resource factors, followed by energy per capita (i.e., population density relative to calories). Therefore, temporal (resource depletion) characteristics appear to have great influence over feeding competition in interaction with gregariousness (Chapman et al., 1995; Isbell & Young, 2002; Macdonald & Johnson, 2015; Waser, 1981). In contrast, clump size did not have a large influence on foraging in the general scenario of our model, implying that resource distribution may have less effect on feeding competition than resource depletability. Although this could be attributable to bias in our model, this appears unlikely, since further work using this model has revealed strong effects of clump size in other circumstances (Ekanayake‐Weber et al., 2024).

In terms of gregariousness itself, both the number of conspecifics and the distance maintained between those conspecifics had an influence on foraging success. In general, being non‐gregarious (i.e., when target neighbors = 0) was the best foraging strategy. This effect was particularly apparent in the fast‐extracting, slow‐renewing scenario. Many solitary‐foraging lemur species are subject to similar ecological pressures that might make gregariousness costly, particularly unpredictable climatic variability, resulting in periodic food scarcity (Dewar & Richard, 2007; Kappeler, 2012; Wright, 1999). In addition, orangutans are unique as the only ape which is neither group‐living nor pair‐living, and their food availability has also been characterized as highly variable and subject to multi‐year climate cycles (Knott, 1998; Vogel et al., 2012, 2017).

However, across a wide range of parameter configurations, agents could pay attention to as many as 10 other agents with little cost, if the target distance was long enough. As has been observed in living animals, large foraging groups can be compensated for by increasing the group spread (e.g., Leighton & Leighton, 1982; van Noordwijk & van Schaik, 1987), or, in fission‐fusion societies, such as those of chimpanzees, spider monkeys, African elephants, and Yunnan snub‐nosed monkeys, groups can fission into smaller foraging parties when the costs of all foraging together are too high (Archie et al., 2006; Chapman et al., 1995; Ren et al., 2012). Alternatively, large groups might forage together when the feeding costs of gregariousness are particularly low, or when the increased predation risk of spreading out imposes a higher fitness cost than feeding competition (Gallagher et al., 2017; Jarman, 1974). Some of the largest, densest primate groups depend on resources that require slow extraction times, such as geladas, who pick grass by hand or dig up underground storage organs, depending on the season (Fashing et al., 2014; Jarvey et al., 2018; Venkataraman et al., 2014).

In the slow‐extracting, fast‐renewing scenario, only the most extreme combination of short target distance and high target neighbors led to very long daily distances traveled. Most group‐living primate species are found close to the equator, in less seasonal environments (but see Jones, 2011), or in habitats that allow them to switch between seasonally available resources and fall‐back foods (Marshall & Wrangham, 2007). Furthermore, primates are able to flexibly adjust their cohesiveness as they move about the “landscape of fear,” increasing density only when predation risk is higher (Coleman & Hill, 2014; Parker et al., 2022). Thus, the overall costs of gregariousness are likely low for most anthropoids, which helps explain the prevalence of group‐living in this clade. Moreover, it was in the slow‐extracting, fast‐renewing scenario that we saw clump size become a significant influence on foraging success. Therefore, it may be that previous ideas which emphasized the influence of patch size on feeding competition, such as the ecological constraints hypothesis (Chapman et al., 1995), were based on this type of parameter space, and failed to consider the wider range of habitats in which resource distribution is less influential.

We also found that variance in energy intake was decreased by gregariousness. Specifically, target distance, which determined the goal maximum distance between individuals, was an important behavioral parameter when it came to variance in intake. As shown by other studies, foraging with other conspecifics can reduce variance in individual intake (e.g., Georgiou et al., 2022). Even in the fast‐extracting, slow‐renewing scenario, shorter target distances reduced variance in intake. This implies that gregariousness might be protective against starvation in harsh environments, such as those that are highly seasonal and where resources are more usurpable (which might have been influential in primate evolution; see Jones, 2011). Similarly, Cody (1971) and Clark and Mangel (1984) hypothesized that information sharing may be most beneficial for birds when resources are highly seasonal and/or quickly depleted. Thus, in response to variable or uncertain conditions, an animal might forage alone for the sake of efficiency, in addition to other adaptations to avoid energy shortfall, such as torpor or internal storage of energy, as seen in certain lemur species (Atsalis, 1999; Fietz & Dausmann, 2007; Schülke & Kappeler, 2003). However, an alternative solution to avoiding energetic shortfall may be to forage in close association with other conspecifics. In the most harsh and marginal environments, we might expect that some species maintain small, extremely spatially cohesive units − especially in lineages where group‐living has already evolved. In fact, many multilevel primate societies exist in unpredictable marginal habitats, e.g., hamadryas baboons, humans, and *Rhinopithecus* (Grueter et al., 2020). This strategy might be particularly effective in combination with other adaptations that improve foraging efficiency, such as more energy‐efficient locomotion (e.g., hominin bipedalism; Leonard & Robertson, 1995; Rodman & McHenry, 1980), gut/digestive adaptations (e.g., segmented stomach; Bell, 1971; Geist, 1974), and memory for goal‐directed travel (as discussed in Janson & Byrne, 2007). By contrast, energy shortfall is not a major challenge in the slow‐extracting, fast‐renewing environment (Figure 9), so there is minimal benefit of foraging gregariously, but the costs are also low. In this case, other costs and benefits may have more influence on the evolution of gregariousness than food characteristics, e.g., how risks of predation or infanticide are impacted by the presence of conspecifics (Bednekoff & Lima, 2004; Crockett & Janson, 2000; Janson & Goldsmith, 1995; Teichroeb et al., 2009).

Beyond explaining the behavior of living taxa, these findings also shed light on what ecological conditions would have favored the evolution of increased gregariousness and, ultimately, the formation of permanent groups in herbivores. Our model indicates two possible evolutionary routes to increased gregariousness: low temporal variability leading to low foraging costs of gregariousness, or, high temporal variability leading to foraging benefits of gregariousness. In the first circumstance, either dietary flexibility (see also Marshall & Wrangham, 2007; Sheppard et al., 2018) or low seasonal variation in calories could have been a major factor in a shift from solitary to gregarious foraging, in the absence of cooperative breeding or cooperative hunting. The species which began to switch from solitary to gregarious foraging may have been generalists, and may have exploited resources that required some degree of processing/handling (Marshall & Wrangham, 2007). When resources require more processing, there is much lower cost to maintaining proximity to conspecifics, because processing slows down the speed of extraction. This applies to both resources like mature leaves and grass, but also nuts, underground storage organs, or tough fruits which require processing (and often specialized adaptations) to be ingested (Marshall & Wrangham, 2007). Also, according to Kleiber's equation (Kleiber & Rogers, 1961) and Kay's threshold (Kay, 1975), the evolution of larger body size reduces the relative amount of energy that an individual requires, and would allow for the greater dependence on slower‐extracting foods (see also Bell, 1971; Geist, 1974; Illius & Gordon, 1992; Jarman, 1974); thus, the increase in body size throughout both primate and ungulate evolution might have lowered the costs of gregariousness (or, conversely, gregariousness led to a selective pressure for larger body sizes; Szemán et al., 2021). In addition, changes to the gut and microbiome may have been pre‐adaptive for sociality in ungulates, because it allowed a shift to food resources that have more fiber content, and therefore, a slower extraction rate (Bell, 1971; Geist, 1974; Illius & Gordon, 1992; Jarman, 1974).

Conversely, very high temporal variability could make gregarious foraging beneficial in reducing the chances of starvation, meaning that it is also possible that high seasonality could lead to the evolution of increased gregariousness. However, our model did not show an effect of target neighbors on this relationship, but in fact, target distance. Therefore, we might not expect these benefits to induce a shift from solitary foraging to gregarious foraging, but instead, these benefits could cause individuals who are already somewhat gregarious to begin maintaining closer proximity to each other as temporal variability increases. As noted above, this may have some relevance to why primates in marginal habitats evolved multi‐level societies, where individuals typically keep in very close proximity within units (Grueter et al., 2020). Interestingly, the evolution of the genus *Homo* was characterized by increasing climate volatility, as well as a rapid expansion into marginal environments (deMenocal, 2011). Hominins have also increasingly utilized time‐intensive food extraction techniques − e.g., investing time into the making of tools in order to obtain and process foods. Together, these factors may have altered the nature of feeding competition for early hominin groups, decreasing costs and increasing the benefits of foraging in close association, and allowing for the evolution of heightened cooperation in our own species.

### 4.1 Limitations and future directions

The agent‐based modeling approach required us to make design choices and simplifications, which unavoidably introduced bias into our study (Williams et al., 2022). In order to recognize and mitigate these biases, we now describe some of the key limitations of the current study. First, the choice of parameters included in the model constrained what factors could be found as influential. During the model design process, we iteratively added more resource parameters in an effort to better match patterns; this included adding the extraction rate and interval of patch regrowth parameters, which were ultimately the most influential in the model. However, we may still be ignoring or misrepresenting some factor from the real world. For example, even when we did add in resource renewal, this process happened “globally,” all at once for the whole landscape. While this may be somewhat analogous to a seasonal fruiting peak, it would have been interesting to also test the possibility of individual patches renewing after being consumed. In addition, we did not explicitly represent predation (or predation risk) as the opposing force to feeding competition in our model. However, agents implicitly utilized predation avoidance strategies when the target neighbors parameter was increased and/or the target distance parameter was decreased. A more detailed model of predation would require detailed representation of predator behavior in addition to mortality and life‐history processes of the “prey,” necessitating a much longer simulation timescale. While this type of model could reveal interesting effects of gregariousness, predation risk, and feeding competition on fitness, it is beyond the scope of the current study.

We also did not allow our foraging agents to have any memory of previously discovered resources, or employ goal‐directed travel (but see Falcón‐Cortés et al., 2019). This represents a significant simplification, since primates are well‐known to travel their home‐ranges non‐randomly (e.g., de Guinea et al., 2021). We did not have the agents employ an optimal foraging strategy (Pyke, 1984), nor could agents avoid (i.e., maintain a distance away from) stronger competitors. We also did not allow for large‐scale dispersal or travel, since the model landscape was limited to approximately 7.9 km^2^. Finally, though we allowed for food resources to vary along several parameters, food was represented only as energy, and each simulation had only one type of food (i.e., characterized by the same mean extraction rate and mean patch quality). Thus, agents did not have the ability to switch between types of resources when one was depleted or usurped (Marshall & Wrangham, 2007), nor did they have to meet requirements for protein or micronutrients which sometimes may have a large effect on the behavior of real animals (Chapman et al., 2012; but see Borries et al., 2022; Ganzhorn et al., 2017).

Future studies have the potential to address many of these limitations, whether by using the current model infrastructure as a starting point, or by developing a new model framework. The model described here is currently being expanded to investigate how group size impacts feeding competition across different food resource scenarios. Rather than imposing a predetermined group size on the agents, we can use gregariousness parameters to manipulate individual behavior and then observe the size of the groups which emerge. We can then investigate (1) what resource characteristics most influence group size, and (2) how group size influences foraging success.

## 5 CONCLUSION

The effects of sociality on feeding competition have long remained poorly understood, leaving the evolutionary pressures on social behavior unclear. With this study, we have demonstrated that the costs and benefits of gregariousness depend greatly on resource characteristics, particularly the depletability of resources. In contrast to previous hypotheses, resource distribution across space is much less influential than these temporal factors. When calories are more consistently available and slower to extract, gregariousness is less costly, whereas when food resources are highly seasonal or very quickly depleted, the costs of being gregarious are higher. However, even in fast‐extracting and/or slow‐renewing circumstances, there may be benefits to gregarious foraging in terms of avoiding energy shortfall. Thus, we are able to better understand the different foraging strategies of group‐living primates and other gregarious herbivores, as well as what ecological conditions might have led to their evolution.

## AUTHOR CONTRIBUTIONS

**Marcy Ekanayake‐Weber:** Conceptualization (equal); formal analysis (equal); funding acquisition (equal); investigation (equal); methodology (equal); project administration (equal); resources (equal); software (equal); supervision (equal); validation (equal); visualization (equal); writing – original draft (equal); writing – review and editing (equal). **Namita Mathew:** Investigation (supporting); software (supporting); visualization (supporting). **Deanna Cunha:** Investigation (supporting); software (supporting). **Nathanael Payen:** Investigation (supporting); software (supporting). **Volker Grimm:** Conceptualization (supporting); methodology (supporting); software (supporting); supervision (equal); validation (supporting); writing – original draft (supporting); writing – review and editing (supporting). **Andreas Koenig:** Conceptualization (supporting); funding acquisition (supporting); project administration (supporting); resources (supporting); supervision (equal); writing – review and editing (equal).

## FUNDING INFORMATION

This work was supported by a National Fellowship from Graduate Women in Science, Inc., awarded to MEW in 2021. This work was also supported by the Dean of the College of Arts and Sciences of Stony Brook University.

## CONFLICT OF INTEREST STATEMENT

The authors have no conflicts of interest.

## Data Availability

All model code, analysis code, and model output data analyzed are openly available in the project repository located at https://github.com/marcysweber/gregariousness.

## APPENDIX 1

### Pattern‐matching data

The model was calibrated and validated using patterns derived from field data for a wide variety of primate species. Two key patterns of primate foraging behavior, daily path length (Vidal‐Cordasco et al., 2020) and activity budget (Kamilar & Cooper, 2013), were derived from recent comparative studies. Though there are methodological concerns with such data (e.g., see Patterson et al., 2014), here they were only used to decide what range of parameter space to focus on within the model, rather than matching exactly the characteristics of any one species. Daily path length for primates was observed to vary from less than 200 meters per day (*Eulemur fulvus fulvus*), up to extremes of 6 km (*Macaca nigra*), 8.6 km (*Papio hamadryas*), and 10 km (*Homo sapiens*), with 97% of species falling between 0.25 and 4.0 km per day (Vidal‐Cordasco et al., 2020). The ratio of time spent moving versus eating also varied widely, from 0.067 to 0.77 (Kamilar & Cooper, 2013). For approximately 85% of primate species (59 out of 70 species for which activity budget data was available), the ratio of time moving: eating was between 0.25 and 0.6 (Kamilar & Cooper, 2013). Using data from preliminary runs, we calibrated our model to focus on the parameter space which generated daily path lengths between 0.25 to 4.0 km and moving: eating ratios between 0.25 to 0.6.

### Model calibration sensitivity analysis

Preliminary analysis of the model tested the relative influence of 15 parameters, listed in Table 1. Five parameters which had extremely low influence were excluded from subsequent (hypothesis‐testing) analyses. These parameters were: standard deviation of patch quality (“qual‐sd”), proportion of patch energy that regrew when regrowth was triggered (“regrowth‐rate”), amount of randomness in agent's random correlated walk (“movement‐noise”), the range at which agents could detect resources (“resource‐detection‐radius”), and the range at which agents could detect other agents (“other‐primate‐detection‐radius”). Note that these parameters cluster in the lower left corner of the Morris Elementary Effects space in Figures OS2.3 and OS2.4.

### Morris elementary effects of variance in intake in alternative scenarios

**TABLE A1:** Parameter ranges and constant settings used to generate the heatmaps presented in Figures 4, 5, 7, and 8.

Figure	Scenario	Parameters of interest	Parameters of interest ranges [start, step, stop]	Output(s) of interest	Constants
4a	General	tgt‐neighbors × tgt‐dist	[“tgt‐dist” [0.5 5 50]] [“tgt‐neighbor” [0 1 11]]	Daily distance traveled	[“abundance” 500,000] [“energy‐per‐capita” 8000] [“extraction‐rate‐sd” 1] [“resource‐tests‐on?” false] [“extraction‐rate‐mean” 7] [“qual‐sd” 1] [“other‐primate‐detection‐radius” 50] [“qual‐mean” 100] [“movement‐noise” 10] [“go‐tests‐on?” false] [“move‐tests‐on?” false] [“resource‐detection‐radius” 50] [“regrowth‐rate” 1] [“group‐recog‐module?” false] [“clump‐size” 500] [“patch‐regrowth‐interval” 1500]
4b	General	Extraction rate × energy per capita	[“extraction‐rate‐mean” [4 0.7 11]] [“energy‐per‐capita” [4000 500 9000]]	Daily distance traveled	[“abundance” 500,000] [“extraction‐rate‐sd” 1] [“resource‐tests‐on?” false] [“qual‐sd” 1] [“tgt‐dist” 25] [“other‐primate‐detection‐radius” 50] [“qual‐mean” 100] [“movement‐noise” 10] [“go‐tests‐on?” false] [“move‐tests‐on?” false] [“tgt‐neighbor” 3] [“resource‐detection‐radius” 50] [“regrowth‐rate” 1] [“group‐recog‐module?” false] [“clump‐size” 500] [“patch‐regrowth‐interval” 1500]
4c and 7a	General	Extraction rate × patch regrowth int	[“extraction‐rate‐mean” [4 0.7 11]] [“patch‐regrowth‐interval” [1000 181 3000]]	Daily distance traveled and variance in intake	[“abundance” 500,000] [“energy‐per‐capita” 8000] [“extraction‐rate‐sd” 1] [“resource‐tests‐on?” false] [“qual‐sd” 1] [“tgt‐dist” 25] [“other‐primate‐detection‐radius” 50] [“qual‐mean” 100] [“movement‐noise” 10] [“go‐tests‐on?” false] [“move‐tests‐on?” false] [“tgt‐neighbor” 3] [“resource‐detection‐radius” 50] [“regrowth‐rate” 1] [“group‐recog‐module?” false] [“clump‐size” 500]
4d	General	tgt‐neighbors × extraction rate	[“tgt‐neighbor” [0 1 11]] [“extraction‐rate‐mean” [4 0.7 11]]	Daily distance traveled	[“abundance” 500,000] [“energy‐per‐capita” 8000] [“extraction‐rate‐sd” 1] [“resource‐tests‐on?” false] [“qual‐sd” 1] [“tgt‐dist” 25] [“other‐primate‐detection‐radius” 50] [“qual‐mean” 100] [“movement‐noise” 10] [“go‐tests‐on?” false] [“move‐tests‐on?” false] [“resource‐detection‐radius” 50] [“regrowth‐rate” 1] [“group‐recog‐module?” false] [“clump‐size” 500] [“patch‐regrowth‐interval” 1500]
4e	General	tgt‐neighbors × patch regrowth int	[“tgt‐neighbor” [0 1 11]] [“patch‐regrowth‐interval” [1000 181 3000]]	Daily distance traveled	[“abundance” 500,000] [“energy‐per‐capita” 8000] [“extraction‐rate‐sd” 1] [“resource‐tests‐on?” false] [“extraction‐rate‐mean” 7] [“qual‐sd” 1] [“tgt‐dist” 25] [“other‐primate‐detection‐radius” 50] [“qual‐mean” 100] [“movement‐noise” 10] [“go‐tests‐on?” false] [“move‐tests‐on?” false] [“resource‐detection‐radius” 50] [“regrowth‐rate” 1] [“group‐recog‐module?” false] [“clump‐size” 500]
4f, 7c	General	tgt‐dist × patch regrowth int	[“tgt‐dist” [0.5 5 50]] [“patch‐regrowth‐interval” [1000 181 3000]]	Daily distance traveled and variance in intake	[“abundance” 500,000] [“energy‐per‐capita” 8000] [“extraction‐rate‐sd” 1] [“resource‐tests‐on?” false] [“extraction‐rate‐mean” 7] [“qual‐sd” 1] [“other‐primate‐detection‐radius” 50] [“qual‐mean” 100] [“movement‐noise” 10] [“go‐tests‐on?” false] [“move‐tests‐on?” false] [“tgt‐neighbor” 3] [“resource‐detection‐radius” 50] [“regrowth‐rate” 1] [“group‐recog‐module?” false] [“clump‐size” 500]
7b	General	tgt‐dist × extraction rate	[“tgt‐dist” [0.5 5 50]] [“extraction‐rate‐mean” [4 0.7 11]]	Variance in intake	[“abundance” 500,000] [“energy‐per‐capita” 8000] [“extraction‐rate‐sd” 1] [“resource‐tests‐on?” false] [“qual‐sd” 1] [“other‐primate‐detection‐radius” 50] [“qual‐mean” 100] [“movement‐noise” 10] [“go‐tests‐on?” false] [“move‐tests‐on?” false] [“tgt‐neighbor” 3] [“resource‐detection‐radius” 50] [“regrowth‐rate” 1] [“group‐recog‐module?” false] [“clump‐size” 500] [“patch‐regrowth‐interval” 1500]
5a	Fast E slow R	tgt‐neighbors × tgt‐dist	[“tgt‐dist” [0.5 5 50]] [“tgt‐neighbor” [0 1 11]]	Daily distance traveled	[“abundance” 500,000] [“energy‐per‐capita” 4500] [“extraction‐rate‐sd” 1] [“resource‐tests‐on?” false] [“extraction‐rate‐mean” 9.5] [“qual‐sd” 1] [“other‐primate‐detection‐radius” 50] [“qual‐mean” 100] [“movement‐noise” 10] [“go‐tests‐on?” false] [“move‐tests‐on?” false] [“resource‐detection‐radius” 50] [“regrowth‐rate” 1] [“group‐recog‐module?” false] [“clump‐size” 500] [“patch‐regrowth‐interval” 2750]
8a	Fast E Slow R	Tgt‐dist × Extraction rate	[“tgt‐dist” [0.5 5 50]] [“extraction‐rate‐mean” [7 0.3 11]]	Variance in intake	[“abundance” 500,000] [“energy‐per‐capita” 4500] [“extraction‐rate‐sd” 1] [“resource‐tests‐on?” false] [“qual‐sd” 1] [“other‐primate‐detection‐radius” 50] [“qual‐mean” 100] [“movement‐noise” 10] [“go‐tests‐on?” false] [“move‐tests‐on?” false] [“tgt‐neighbor” 3] [“resource‐detection‐radius” 50] [“regrowth‐rate” 1] [“group‐recog‐module?” false] [“clump‐size” 250] [“patch‐regrowth‐interval” 2750]
8b	Fast E slow R	tgt‐dist × patch regrowth int	[“tgt‐dist” [0.5 5 50]] [“patch‐regrowth‐interval” [2500 45 3000]]	Variance in intake	[“abundance” 500,000] [“energy‐per‐capita” 4500] [“extraction‐rate‐sd” 1] [“resource‐tests‐on?” false] [“extraction‐rate‐mean” 9.5] [“qual‐sd” 1] [“other‐primate‐detection‐radius” 50] [“qual‐mean” 100] [“movement‐noise” 10] [“go‐tests‐on?” false] [“move‐tests‐on?” false] [“tgt‐neighbor” 3] [“resource‐detection‐radius” 50] [“regrowth‐rate” 1] [“group‐recog‐module?” false] [“clump‐size” 500]
5b	Fast E Slow R	Clump size × patch quality	[“qual‐mean” [25 11 150]] [“clump‐size” [1 90 1000]]	Daily distance traveled	[“abundance” 500,000] [“energy‐per‐capita” 4500] [“extraction‐rate‐sd” 1] [“resource‐tests‐on?” false] [“extraction‐rate‐mean” 9.5] [“qual‐sd” 1] [“other‐primate‐detection‐radius” 50] [“movement‐noise” 10] [“go‐tests‐on?” false] [“move‐tests‐on?” false] [“tgt‐neighbor” 3] [“resource‐detection‐radius” 50] [“regrowth‐rate” 1] [“group‐recog‐module?” false] [“tgt‐dist” 25] [“tgt‐neighbor” 3]
5c	Slow E fast R	tgt‐neighbors × tgt‐dist	[“tgt‐dist” [0.5 5 50]] [“tgt‐neighbor” [0 1 11]]	Efficiency	[“abundance” 500,000] [“energy‐per‐capita” 9000] [“extraction‐rate‐sd” 1] [“resource‐tests‐on?” false] [“extraction‐rate‐mean” 5.5] [“qual‐sd” 1] [“other‐primate‐detection‐radius” 50] [“qual‐mean” 100] [“movement‐noise” 10] [“go‐tests‐on?” false] [“move‐tests‐on?” false] [“resource‐detection‐radius” 50] [“regrowth‐rate” 1] [“group‐recog‐module?” false] [“clump‐size” 500] [“patch‐regrowth‐interval” 1500]
5d	Slow E fast R	Clump size × patch quality	[“qual‐mean” [25 11 150]] [“clump‐size” [1 90 1000]]	Daily distance traveled	[“abundance” 500,000] [“energy‐per‐capita” 9000] [“extraction‐rate‐sd” 1] [“resource‐tests‐on?” false] [“extraction‐rate‐mean” 5.5] [“qual‐sd” 1] [“tgt‐dist” 25] [“other‐primate‐detection‐radius” 50] [“movement‐noise” 10] [“go‐tests‐on?” false] [“move‐tests‐on?” false] [“tgt‐neighbor” 3] [“resource‐detection‐radius” 50] [“regrowth‐rate” 1] [“group‐recog‐module?” false] [“patch‐regrowth‐interval” 1500]

*Note*: All parameters except the two presented on the *x*‐ and *y*‐axis of the heatmaps (“params of interest”) were held constant at an intermediate value. Scenario abbreviations: general, full range of depletability parameters; fast E, fast extracting; slow E, slow extracting; fast R, fast regrowth (short patch regrowth interval); slow R, slow regrowth (long patch regrowth interval).
